# Asymmetric Mass Transport in Polybromide Ionic Liquids and Its Impact on Dual‐Plating Zinc Bromine Batteries

**DOI:** 10.1002/advs.202522078

**Published:** 2026-02-08

**Authors:** Sung Il Kim, Kyungjae Shin, Heejung Kang, Seok Hee Han, Hee‐Tak Kim, Taek Dong Chung

**Affiliations:** ^1^ Department of Chemistry Seoul National University Seoul Republic of Korea; ^2^ Department of Chemical & Biomolecular Engineering Korea Advanced Institute of Science and Technology (KAIST) Daejeon Republic of Korea; ^3^ Department of Materials Science and Engineering Massachusetts Institute of Technology Seok Hee Han, Cambridge Massachusetts USA; ^4^ Advanced Institutes of Convergence Technology Suwon‐Si Gyeonggi‐do Republic of Korea

**Keywords:** electrochemical impedance spectroscopy, energy storage, ionic liquid, mass transport, zinc–bromine battery

## Abstract

Zinc‐halogen batteries (ZHBs) offer a safer, cost‐effective alternative to lithium‐ion batteries, leveraging abundant zinc resources and high energy density. Among ZHBs, dual‐plating zinc bromine batteries (ZBBs) utilizing ionic liquid (IL)‐forming bromine complexing agents (BCAs) exhibit enhanced performance by minimizing halogen crossover and enabling high conductivity via Grotthuss‐type halide transport. In this study, the electrochemical impedance of the bromide redox reaction in the presence of 1‐ethyl‐1‐methylpyrrolidinium bromide (MEPBr), an IL‐forming BCA, was analyzed. Potentiodynamic *operando* impedance measurements revealed pronounced asymmetry in mass transport impedance between polybromide ionic liquid (PBIL) formation and dissolution. This asymmetry significantly influenced the potential and impedance trends during galvanostatic cycling of dual‐plating ZBBs. During charging, facilitated Br^−^ transport lowered the positive electrode impedance, resulting in minimal positive electrode overpotential even at high current densities. In contrast, during discharging, PBIL dissolution at the positive electrode exhibited large overpotential at high current densities due to the relatively sluggish internal mass transport of Br_2n+1_^−^. Furthermore, similar asymmetry was observed across various IL‐forming BCAs, indicating that the mass transport disparity is an intrinsic property of PBIL rather than limited to MEPBr. These findings provide new insights into PBIL mass transport dynamics and their impact on high‐current‐density operation in dual‐plating ZBBs.

## Graphical abstract

This study elucidates mass transport dynamics within polybromide ionic liquids in dual‐plating zinc–bromine batteries using operando impedance spectroscopy. The findings reveal that sluggish polybromide transport during dissolution contrasts with rapid bromide transport during charging. This intrinsic asymmetry causes severe discharge polarization, dictating the high‐rate performance limitations and offering guidelines for optimizing electrolyte design.

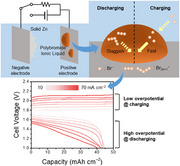

## 1 Introduction

Aqueous zinc‐based batteries are considered a high‐safety alternative to lithium‐ion batteries due to their non‐flammable nature and use of abundant zinc resources, making them suitable for applications where safety and cost are paramount [1, 2, 3]. Among zinc‐based battery chemistries, zinc‐halogen batteries (ZHBs) using either iodine or bromine are particularly promising due to their high specific capacity and moderate operating voltages (1.2 V for zinc–iodine and 1.8 V for zinc–bromine) [4, 5, 6, 7, 8, 9, 10, 11, 12, 13, 14]. These characteristics enable energy‐dense storage while avoiding the safety risks associated with lithium‐based systems.

However, zinc–halogen batteries face significant challenges, primarily related to self‐discharge [15]. During discharge, reactive polyhalides (e.g., I_3_^−^ or Br_3_^−^) form at the positive electrode, which can diffuse toward the zinc negative electrode and react with it, causing rapid self‐discharge [6, 16, 17, 18]. One approach to mitigate this issue is to utilize halogen complexing agents capable of limiting halide diffusion toward the negative electrode [19, 20, 21, 22, 23]. It was found that certain quaternary alkyl ammonium cations (QA^+^) can locally form an immiscible solid or liquid phase on the positive electrode surface, which prevents the polyhalides from diffusing towards the aqueous phase. This type of battery was termed a dual‐plating zinc‐halogen battery, as immiscible QAX_2n+1_ phase and solid Zn are deposited on the positive and negative electrode surfaces, respectively, during charging, and are removed during discharging [24, 25, 26, 27].

In case of complexing agents that form solids, the dual‐plating battery showed low conductance, which is due to the sluggish charge and mass transport of crystallized QAX_2n+1_ on the positive electrode surface [20, 24]. On the other hand, for complexing agents forming an ionic liquid (IL) phase, the dual‐plating batteries demonstrated superior conductance, which is thought to be due to the Grotthuss‐type halide transport in the IL phase [24]. This mechanism, involving the transfer of halogen species through successive bonding and release events—analogous to the proton transport mechanism in water—is believed to be the primary contributor to the high conductance observed in the polyhalide ionic liquid, enabling the charging and discharging of ZHBs at exceptionally high current densities [28, 29, 30, 31, 32, 33, 34, 35, 36]. However, the precise physicochemical nature of electrochemically formed polyhalide ILs remains a subject of debate and is yet to be fully elucidated. This unresolved issue is thought to be closely related to one of the challenges in ZHB development—the pronounced cell polarization observed during discharge, in contrast to the relatively small polarization during charge [37, 38]. A clearer understanding of the mass transport in polyhalide ILs is therefore critical to improving the overall performance of ZHBs.

In this study, we investigated the electrochemical properties, with a particular focus on the transport properties of bromine species in the presence of polybromide ionic liquid (PBIL), which forms during electrochemical oxidation in the presence of 1‐ethyl‐1‐methylpyrrolidinium bromide (MEPBr), an IL‐forming bromine complexing agent (BCA). Using cyclic voltammetry (CV) and Fourier Transform Electrochemical Impedance Spectroscopy (FTEIS) [39, 40, 41, 42, 43, 44], we identified a significant disparity in mass transport impedance between PBIL formation and dissolution, corresponding to the charging and discharging processes of ZBB. The impact of mass‐transport asymmetry on ZBB performance was further evaluated using *operando* galvanostatic FTEIS. The results revealed that this asymmetry gives rise to high overpotentials during discharging, whereas overpotentials during charging remain relatively small. These findings deepen the understanding of PBIL mass‐transport behavior and highlight that the performance of ZBBs employing IL‐forming BCAs is strongly governed by the transport dynamics of bromine species.

## 2 Results and Discussion

### 2.1 Plating and Dissolution Dynamics of the Polybromide Ionic Liquid Phase in Dual‐Plating Zinc–Bromine Batteries

Typical dual‐plating ZBBs operate through the following reactions for energy storage and release:

(1) $$Br_{2}\;\left(\mathrm{aq}\right)+2e^{-}⇌2Br^{-}\left(\mathrm{aq}\right)\to \left(E^{0}=1.08\;V\;\mathrm{vs}.\;\mathrm{SHE}\right)$$

(2) $$Zn^{2+}\left(\mathrm{aq}\right)+2e^{-}⇌\mathrm{Zn}\;\left(s\right)\to \left(E^{0}=-0.76\;V\;\mathrm{vs}.\;\mathrm{SHE}\right)$$

(3) $$Zn^{2+}\left(\mathrm{aq}\right)+2Br^{-}\left(\mathrm{aq}\right)⇌\mathrm{Zn}\;\left(s\right)+Br_{2}\;\left(\mathrm{aq}\right)\to \left(E_{\mathrm{cell}}=1.84\;V\right)$$

During charging, Br_2_ that accumulates at the positive electrode surface readily reacts with bromide ions in the electrolyte to form polybromides (Br_2n+1_^−^):

(4) $$Br^{-}\left(\mathrm{aq}\right)+\mathrm{nB}r_{2}\left(\mathrm{aq}\right)⇌{\mathrm{Br}}_{2n+1}^{-}\left(\mathrm{aq}\right)$$

Eventually, the polybromide complex keeps oxidized bromine highly soluble in the aqueous electrolyte; as it diffuses toward the negative electrode, it reacts with solid zinc and causes self‐discharge.

BCAs can stabilize polybromide anions by forming immiscible ionic solids or liquids on the positive electrode surface. In the presence of the ionic‐liquid‐forming BCA, MEPBr, a PBIL phase is generated on the positive electrode: [9]

(5) $${\mathrm{Br}}_{2n+1}^{-}\left(\mathrm{aq}\right)+\mathrm{ME}P^{+}\left(\mathrm{aq}\right)⇌\mathrm{MEPB}r_{2n+1}\left(\mathrm{IL}\right)$$

PBILs produced from MEP^+^ or other BCAs are highly conductive, likely due to the Grotthuss‐type transport of bromine species—namely Br^−^, Br_2_, and Br_2n+1_^−^ [30, 31, 32, 33, 45]. Also, it is known that most of the Br_2_ exists as Br_2n+1_^−^ inside the PBIL due to the stabilization of BCAs [20].

**FIGURE 1:**
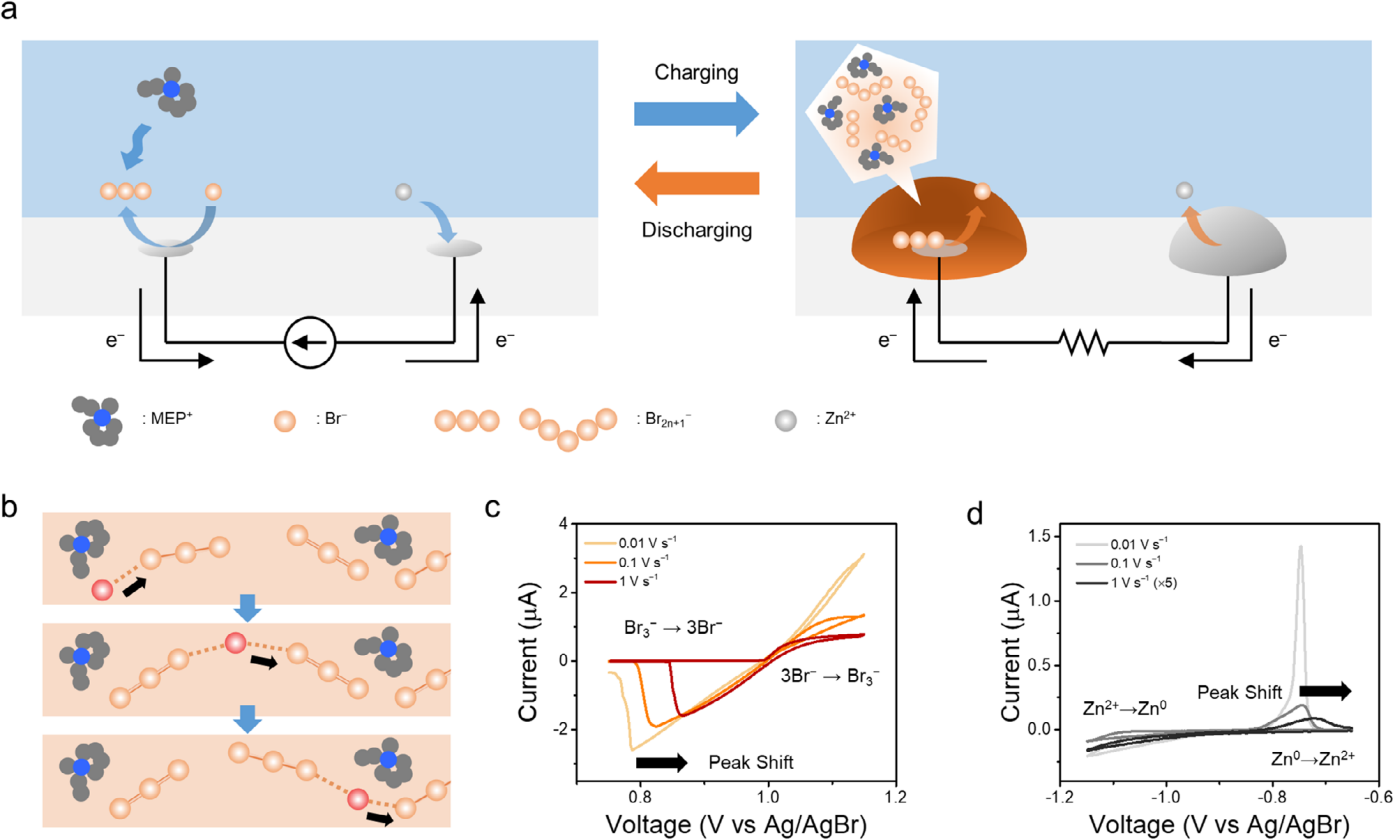
Schematic illustration of (a) a dual‐plating ZBB, showing the processes occurring during charging and discharging, and (b) the Grotthuss‐type transport mechanism within the PBIL phase, illustrating the successive bond formation and cleavage. Cyclic voltammograms recorded from a 10 µm Pt UME immersed in 0.5 M ZnSO_4_ + 0.25 M MEPBr at varying scan rates, showing (c) the PBIL formation/dissolution potential range and (d) the zinc plating/dissolution potential range. Arrows indicate the shift in dissolution peak position as the scan rate increases. The current for 1 V s^−1^ in (d) has been amplified 5 times for clarity.

The electrochemical processes during charging and discharging of dual‐plating ZBB are schematically illustrated in Figure 1a. During charging, PBIL gets “plated” on the positive electrode and solid zinc on the negative electrode. During discharge, both the PBIL phase and solid zinc are removed from the electrode surfaces. Generally, ZBBs utilizing IL‐forming BCAs are known to exhibit superior rate capability compared to those using solid‐forming agents [26], a property largely attributed to the high ionic conductivity of the PBIL phase. The specific transport mechanism underlying this high conductivity is conceptually visualized in Figure 1b. Within the PBIL, bromine species are believed to be transported via a Grotthuss‐type hopping mechanism. Unlike the physical diffusion (vehicle mechanism), this process involves charge transfer through successive bond formation and cleavage events, enabling rapid transport despite the high viscosity of the ionic liquid.

Despite these advantageous transport properties, however, dual‐plating ZBBs often exhibit a pronounced asymmetry between charge and discharge performance, as noted in the introduction. To elucidate the physicochemical origin of this asymmetry, we first investigated the fundamental plating and dissolution dynamics of the PBIL and zinc phases. As a primary step, the current response obtained from a 10 µm Pt ultramicroelectrode (UME) was analyzed to track these processes (Figure 1c,d, Figure S5a) [46].

Figure 1c,d shows the current responses for PBIL and zinc plating/dissolution, respectively, at varying scan rates (full potential range CV in Figure S5a; non‐amplified version in Figure S5b). More material was deposited at lower scan rates because the electrode experienced longer plating times at reduced scan rates (Figure S5b). When observing the deposition potential range (magnified in Figures S5c,d), a current increase upon scan direction reversal was observed for both PBIL and zinc, except at 1 V s^−1^. Typically, during electrodeposition on a UME, the reverse scan current is higher due to the presence of nucleation overpotential and the increased electrode surface area resulting from the deposition process [47]. At a scan rate of 1 V s^−1^, the absence of a current increase is likely attributed to the insufficient formation of PBIL and solid zinc on the electrode surface, as well as the contribution of capacitive current. In the presence of polyhalide ionic liquids, it has been suggested that Grotthuss‐type transport enables the rapid oxidation of halide ions upon reaching the IL surface, giving rise to metal‐deposition‐like voltammograms [24, 28].

However, the dissolution peak position trends differed between PBIL and solid zinc: the peak for PBIL shifted toward lower overpotential with increasing scan rate, whereas the dissolution peak for solid zinc shifted toward higher overpotential. In typical electroplating systems, dissolution of electroplated species requires no mass transport, and they are expected to be removed immediately once sufficient charge transfer overpotential is applied. As the scan rate increases, the rapid potential sweep causes the peak to shift toward higher overpotential, consistent with the observed behavior for zinc [48, 49]. Nevertheless, this explanation does not account for the dissolution peak shift of PBIL. Unlike the “metal electrodeposition‐like” current response observed during PBIL plating, the observed dissolution peak shift suggests the presence of an internal mass transport limitation within the PBIL phase during dissolution.

**FIGURE 2:**
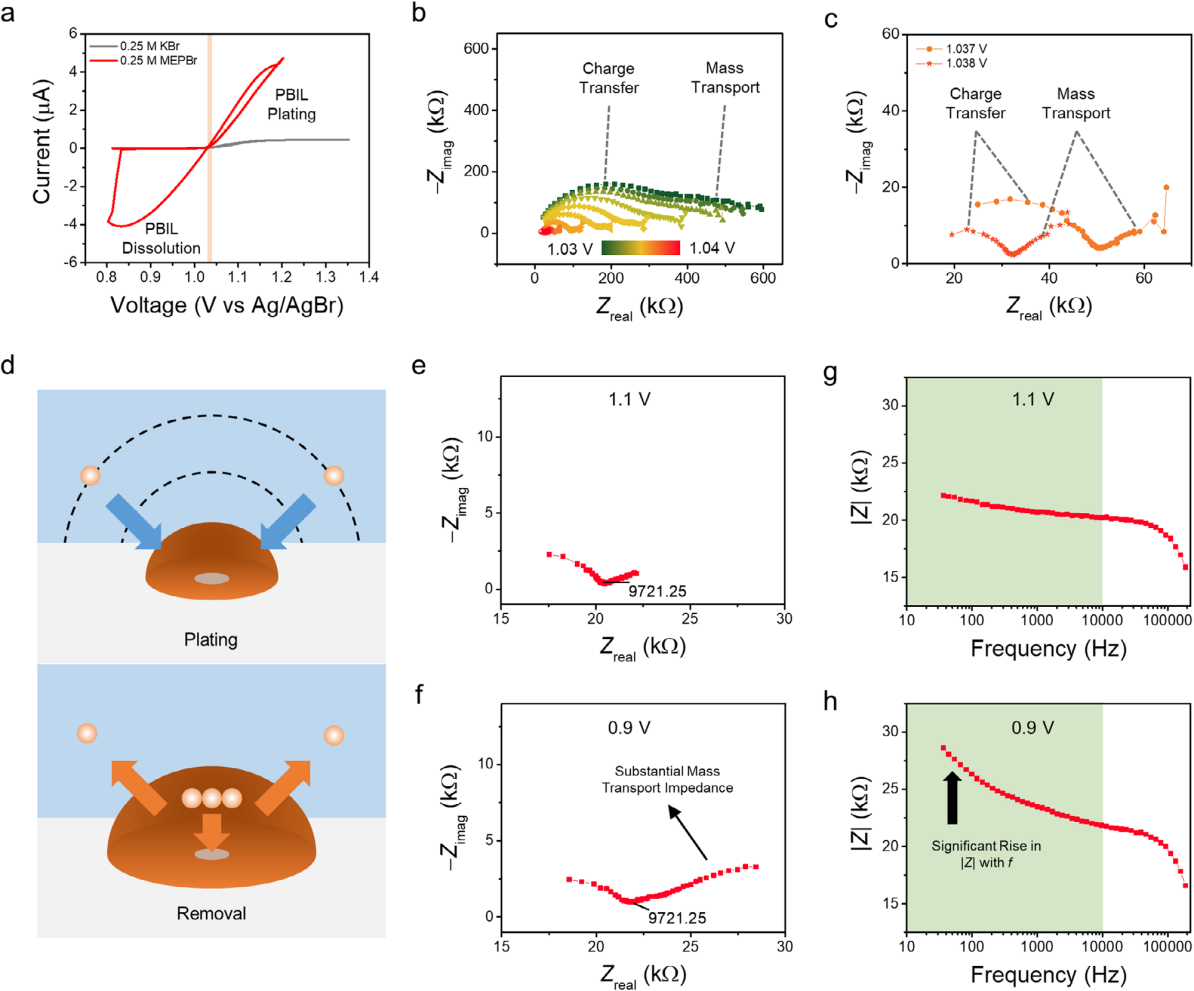
(a) Cyclic voltammograms recorded at 10 mV s^−1^ for a 10 µm Pt UME in solutions of 0.25 M KBr + 0.5 M H_2_SO_4_ (gray) and 0.25 M MEPBr + 0.5 M H_2_SO_4_ (red). (b) Nyquist plots showing the transition for a 0.25 M MEPBr solution during potential cycling from 1.03 to 1.04 V, corresponding to the shaded orange region in (a). The plots display impedance data for frequencies ranging from 200 kHz to 100 Hz. (c) Nyquist plots obtained at 1.037 and 1.038 V, corresponding to the impedance spectra after PBIL formation. (d) Schematic illustration showing the mass transport of associated chemical species during PBIL plating and removal. The dashed line denotes the concentration contour of Br^−^. Nyquist plots obtained 20 s after the application of (e) 1.1 V and (f) 0.9 V, with (f) recorded after 1000 s of PBIL formation at 1.1 V. Bode plots corresponding to the conditions in (e) and (f) are presented in (g) and (h), respectively, with the shaded green region indicating the frequency range associated with mass transport impedance. The impedance plots are shown for frequencies ranging from 200 kHz to 100 Hz for (b,c) and down to 30 Hz for (e–h).

Multisine FTEIS, a rapid impedance measurement technique [41, 44], was utilized to investigate the mass transport properties of bromine species during PBIL plating and dissolution [24, 26, 29]. The fundamentals of EIS measurements are provided in Supporting Information: Note S1 and Figure S6. Figure 2a presents the CV recorded from a Pt UME immersed in 0.25 M MEPBr + 0.5 M H_2_SO_4_, while simultaneously monitoring the impedance by FTEIS. The CV for MEPBr exhibits a pronounced increase in anodic current along with a prominent dissolution peak, consistent with the behavior shown in Figure 1 and other references [30, 31], whereas the CV for KBr instead of MEPBr displays the typical steady‐state current response characteristic of UMEs. This current amplification in the MEPBr case can be attributed to rapid shuttling of Br^−^ toward the electrode surface via Grotthuss‐type transport in the PBIL [29].

The transition of the Nyquist plot during PBIL formation (1.03 to 1.04 V) is presented in Figure 2b, and plots obtained at 1.037 and 1.038 V, corresponding to the impedance spectra immediately after PBIL formation, are shown in Figure 2c. At 1.03 V, the Nyquist plot exhibits a high‐frequency semicircle followed by a depressed semicircle at lower frequencies, characteristic impedance spectra when using a UME that can be modeled with a Randles circuit incorporating a spherical diffusion Warburg (Figure S6b) [44]. The high‐frequency semicircle corresponds to the charge transfer resistance (or polarization resistance for *E* ≠ *E*_eq_) and double layer capacitance (*C*_dl_ or *Q*_dl_ for non‐ideal capacitance), while the low‐frequency arc is associated with radial diffusion towards the UME. However, as the PBIL phase forms, the Nyquist plot undergoes significant changes: the overall impedance is markedly reduced, and the characteristic low‐frequency arc transitions into a straight line (Figure 2c). Because the PBIL phase contains a high concentration of Br^−^ and Br_2n+1_^−^ [29], its formation reduces both charge‐transfer resistance and mass‐transport impedance. The origin of the altered mass‐transport impedance after PBIL formation is discussed in detail in Supporting Information: Note S2. The smooth transition of the Nyquist plot between 1.03 and 1.04 V (Figure 2b) indicates that the high‐frequency semicircle and the low‐frequency line observed after PBIL formation (Figure 2c) correspond to charge transfer and mass transport, respectively. Accordingly, the impedance spectra obtained in the presence of PBIL on the electrode surface were fitted throughout this study using the Randles circuit in Figure S6b.

The internal mass transport impedance of the PBIL was further investigated during its formation and dissolution at overpotentials (*η*) of approximately ±0.1 V (Figure 2d–h). Figure S8 presents the current response and Figure S9 shows optical microscope images during PBIL formation and removal. As shown in the optical microscope image in Figure S9, an oily orange phase forms upon the oxidation of bromide in the presence of MEP^+^. Although this phase was previously denoted as PBIL, Raman spectroscopy was employed to confirm the presence of polybromide ions. Experimental details and further discussion are provided in Supporting Information: Note S3.

Figure 2d illustrates the mass transport of Br^−^ and Br_2n+1_^−^ during PBIL formation and dissolution. At first glance, one would expect the mass transport impedance to be larger during PBIL formation rather than dissolution. However, comparison of the Nyquist plots in Figure 2e,f reveals that the mass transport impedance during PBIL dissolution at 0.9 V is significantly larger than that during PBIL formation at 1.1 V, despite the charge‐transfer semicircles being similar. A comparison of the Bode plots (Figure 2g,h) further emphasizes the stark contrast in mass transport impedance.

**FIGURE 3:**
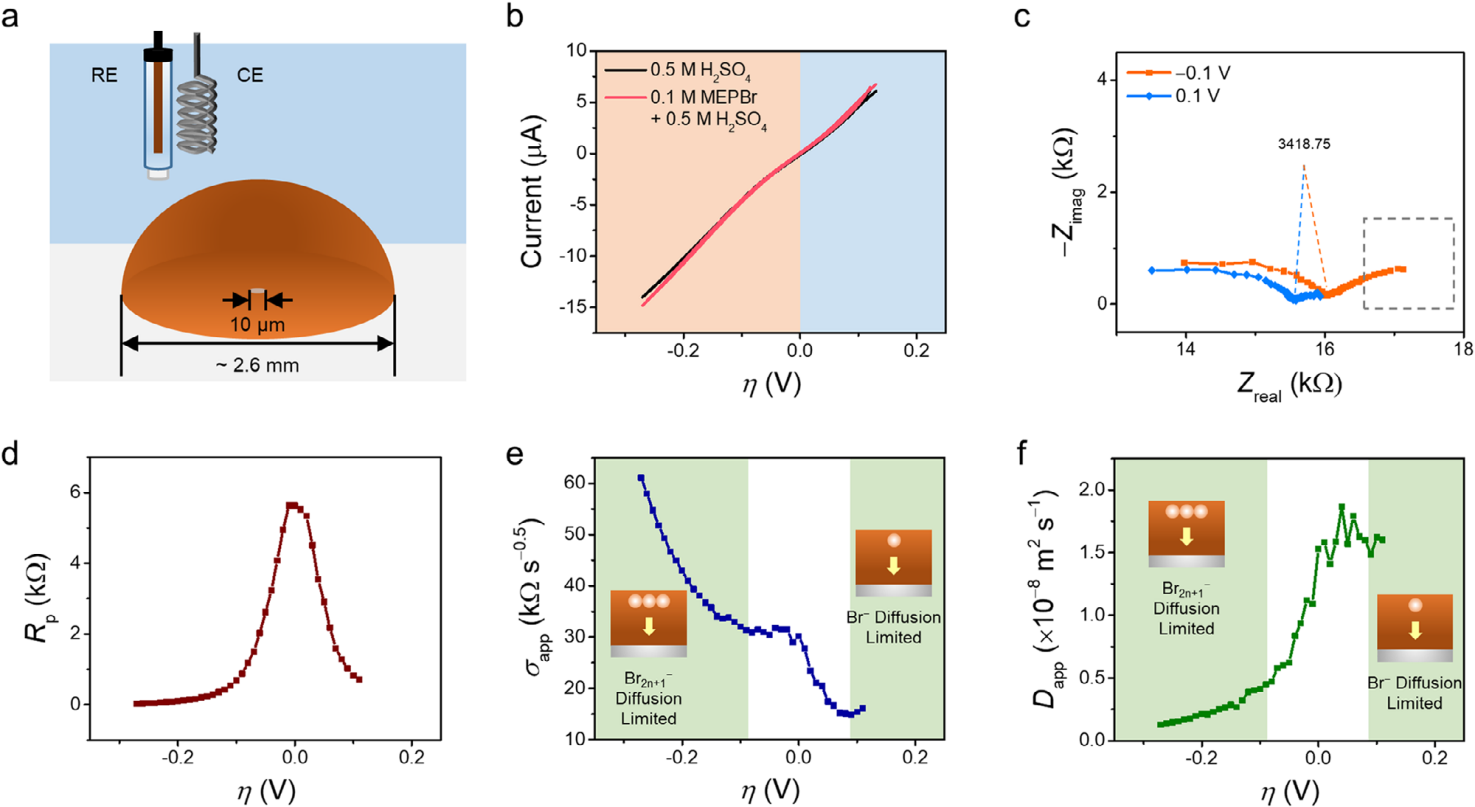
(a) Graphic of the electrochemical experimental setup with 5 µL of PBIL placed on a 10 µm Pt UME. (b) Cyclic voltammograms plotted against overpotential (*η*) for the PBIL‐placed UME immersed in 0.5 M H_2_SO_4_ (black) and 0.1 M MEPBr + 0.5 M H_2_SO_4_ (pink), recorded at a scan rate of 10 mV s^−1^. The shaded blue and orange regions indicate the oxidation and reduction potential ranges, respectively. (c) Nyquist plots obtained at |*η*| = 0.1 V for 0.5 M H_2_SO_4_ during potential cycling in (b). The Nyquists are shown for frequencies ranging from 150 kHz to 30 Hz. (d) The polarization resistance (*R*_P_), (e) apparent Warburg coefficient (*σ*_app_), and (f) apparent diffusion coefficient (*D*_app_) as functions of overpotential, with the shaded green region representing the mass transport regime governed solely by either Br^−^ or Br_2n+1_^−^.

For further investigation of the asymmetric mass transport properties seen in Figure 2, 5 µL of PBIL was placed on a 10 µm‐diameter Pt UME (Figure 3a). The PBIL phase, with a diameter approximately 250 times larger than that of the UME, allows us to assume that the PBIL phase acts as a “bulk” relative to the UME. To confirm that the current response is unaffected by the presence of Br^−^ ions in the aqueous phase—and thus that the PBIL indeed behaves as a bulk phase—CVs were recorded with PBIL‐coated Pt UMEs immersed in two different aqueous solutions: 0.5 M H_2_SO_4_ and 0.1 M MEPBr + 0.5 M H_2_SO_4_ (Figure 3b) The voltammograms are plotted versus overpotential to account for shifts in the equilibrium potential caused by variations in the [Br^−^]_aq_. The two voltammograms show minimal differences, confirming that the PBIL functions as a bulk phase relative to the UME; the movement of Br^−^ as well as MEP^+^ across the aqueous/ionic liquid interface does not alter the electrochemical signal. In addition, the voltammograms resemble those of an irreversible electrochemical system (i.e., charge‐transfer limited) rather than the sigmoidal response typically observed for a reversible system (i.e., mass‐transport limited) when using a UME [50].

When analyzing cyclic voltammograms, it is difficult to obtain kinetic parameters in the mass‐transport‐limited regime or mass‐transport parameters in the kinetically limited regime, since the current primarily reflects the dominant process in each regime. By utilizing FTEIS, however, we were able to quantify both kinetic and mass‐transport parameters across the entire potential range, as the AC response was recorded at each potential bias during cycling. This capability is particularly important because the voltammogram in Figure 3b exhibits charge‐transfer‐limited behavior, in which mass‐transport parameters cannot be extracted without FTEIS.

Two representative Nyquist plots obtained at *η* = ±0.1 V during voltage cycling in Figure 3b using FTEIS are shown in Figure 3c. Similar to the impedance behavior observed earlier (Figure 2), the mass transport impedance was significantly smaller at 0.1 V compared to −0.1 V, while the charge transfer arc remained similar in size. Since the PBIL phase acts as a bulk phase, this difference in mass transport rate can be attributed solely to the difference within the PBIL phase. The transition of Nyquist plots for 0.5 M H_2_SO_4_ and 0.1 M MEPBr + 0.5 M H_2_SO_4_ are shown in Figure S13a,b, respectively. In both cases, the mass transport impedance is noticeably smaller at oxidizing potentials. This difference in mass transport impedance cannot be discerned from the voltammograms, as the current values at *η* = ±0.1 V are approximately similar, around 5 µA.

In order to fit the impedance data, the mathematical expression of spherical Warburg versus overpotential was derived in Supporting Information: Note S4 and is provided in Equations S4–S6. Here, we assume that diffusion, rather than migration, serves as the primary transport mechanism for bromine species, given that the high charge density of the PBIL phase effectively neutralizes the internal electric field [30, 32]. Because the full impedance expression is highly complex, it was simplified for practical fitting by introducing two assumptions: (1) the concentrations, and (2) the square roots of the diffusion coefficients of Br^−^ and Br_2n+1_^−^ are similar in scale. As Br_2n+1_^−^ is a collective label for the entire polybromide series, speaking of its “concentration” can be misleading. What matters electrochemically is the amount of oxidized bromine available for reduction. We therefore define an equivalent polybromide concentration

(6)

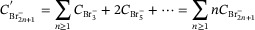

which weights each anion by the number of Br_2_ units it contains. Throughout the following discussion, 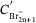 is used when regarding the “concentration of Br_2n+1_^−^”. Likewise, the corresponding “diffusion coefficient” denotes the weighted apparent diffusion coefficient of the Br_2n+1_^−^ ensemble.

When applying the assumption above, for *η*>>0, the contribution of *Z*_W,sph,O_ can be neglected, leaving *Z*_W,sph,R_ to represent the overall mass transport impedance (and vice versa for *η*<<0, where only *Z*_W,sph,O_ is considered; see Supporting Information: Note S4). Under these conditions, the simplified form of the mass transport impedance becomes:

(7) $$Z_{W,\mathrm{sph}}\approx \frac{\sigma }{\sqrt{j\omega }+\frac{\sqrt{D}}{r_{0}}}$$

Here, *D* represents the diffusion coefficient of species O (Br_2n+1_^−^) for *η*<<0 and species R (Br^−^) for *η*>>0, while *σ* is given by Equation S8 for *η*<<0 and Equation S10 for *η*>>0. For simplicity in equivalent circuit model fitting, the mass transport component of the Nyquist plots was fitted to Equation (7) across the entire potential range using two parameters: *σ* and *D*, with the UME radius fixed at *r*_0_ = 5×10^−6^ m. To validate the assumption of comparable concentrations, a simple chronocoulometric and potentiometric method was employed (described in Supporting Information: Note S5). The concentrations of Br^−^ and Br_2n+1_^−^ were found to be 7±3 and 6.4±0.3 M respectively, confirming that they are of similar magnitude.

The Nyquist plots obtained during potential cycling in 0.5 M H_2_SO_4_ were fitted to an equivalent circuit model shown in Figure S6b, with the fitting results of representative Nyquist plots presented in Figure S15. Figure 3d–f shows the fitting results of the Nyquist plots obtained during potential cycling in Figure 3b. To note, the potential was cycled up to *η* = 0.1 V, as this potential sufficiently satisfies the condition of *η* >> 0. Nyquist plots beyond this range deviated from the Randles circuit model, likely due to relatively long term changes in bulk concentration of the PBIL caused by ongoing reaction. This is also evident on the cyclic voltammogram, as cycling up to higher overpotentials showed a current hysteresis (Figure S16). The rationale for this potential range selection is provided in Supporting Information: Note S6.

The polarization resistance *R*_p_ versus *η* curve (Figure 3d) exhibits a typical charge transfer‐limited behavior, showing a maximum near *E*_eq_ [51]. For a mass transport‐limited (reversible) system, the *R*_p_ shows a minimum, rather than maximum, near *E*_eq_, because the rapid charge transfer causes depletion of reduced species at the electrode surface when *η*>0, and similarly, depletion of oxidized species when *η*<0. Thus, the observation of a maximum in *R*_p_ at *E*_eq_ indicates that, relative to charge transfer, mass transport is sufficiently fast such that the supply of reactants and removal of products do not limit the overall charge‐transfer process. Furthermore, the symmetric behavior around *η* = 0 suggests that the intrinsic charge transfer kinetics of the redox reaction on the electrode surface is symmetric.

The apparent Warburg element (*σ*_app_) and the apparent diffusion coefficient (*D*_app_) as functions of overpotential are shown in Figure 3e,f respectively, where the shaded green regions indicate the potential range in which the Warburg impedance is dominated by either *Z*_W,sph,R_ or *Z*_W,sph,O_—that is, where mass transport impedance is governed solely by the diffusion of either Br^−^ or Br_2n+1_^−^ (see Supporting Information: Note S6). The term “apparent” is used rather than diffusion coefficient because the Warburg expression used for fitting is strictly valid only for |*η*|>>0 (fitting of the charge‐transfer process is only minorly affected by the choice of Warburg model, so the term apparent was not applied for *R*_p_). However, applying the model across the entire potential range is advantageous as it enables a comprehensive visualization of the transition in mass transport impedance properties.

As the system operates in a charge‐transfer‐limited regime, the Warburg coefficient can be further simplified, as detailed in Equations S11 and S12 in Supporting Information: Note S4:

(8) $$\sigma =\left\{\begin{array}{c} \frac{RT}{n^{2}F^{2}A\sqrt{D_{O}}\left(\alpha C_{O}\left(0\right)\right)},\;\eta \ll 0\\ \frac{RT}{n^{2}F^{2}A\sqrt{D_{R}}\left(\left(1-\alpha \right)C_{R}\left(0\right)\right)},\;\eta \gg 0 \end{array}\right.\;$$

Under reducing conditions, the Warburg coefficient depends on the diffusion coefficient and surface concentration of Br_2n+1_^−^, while under oxidizing conditions, it is governed by those of Br^−^. The *σ*_app_ versus *η* (Figure 3e) shows a drastic decrease when the current polarity switches from reducing to oxidizing, indicating that the overall mass transport required for current flow is relatively facilitated under oxidizing conditions.

Also, the apparent diffusion coefficient versus overpotential (Figure 3f) reveals a clear disparity in diffusion coefficients between Br^−^ and Br_2n+1_^−^. Specifically, the diffusion coefficients of Br^−^ and Br_2n+1_^−^, extracted from the endpoints of the potential range, were calculated as 1.60 × 10^−8^ and 1.27 × 10^−9^ m^2^ s^−1^, respectively. Notably, the value for Br^−^ is approximately an order of magnitude higher than its value in aqueous solution (2.1 × 10^−9^ m^2^ s^−1^) and is comparable to that of protons in water (9.3 × 10^−9^ m^2^ s^−1^). Since protons are known to be transported via a Grotthuss mechanism, it is possible that bromides are also transported in a similar manner, as suggested by previous studies [29, 30, 34, 52]. This observation remains consistent with the assumption ($\sqrt{D_{O}}∼\sqrt{D_{R}}$, i.e., the two values are of a similar order of magnitude) utilized in the derivation in Supporting Information: Note S4, as the square roots of the two diffusion coefficients differ by a factor of approximately 3.5.

In contrast, the diffusion coefficient of Br_2n+1_^−^ is an order of magnitude lower than that of Br^−^ and instead falls within the range of inorganic ions in water (1–2 × 10^−9^ m^2^ s^−1^). This may serve as evidence that Br_2n+1_^−^ does not diffuse via a Grotthuss mechanism inside the PBIL. Polybromide anions are essentially complexes of Br^−^ coordinated with Br_2_ molecules (Equation 6), and from a mechanistic standpoint, exchanging a neutral Br_2_ unit between neighboring coordination environments is more plausible than a concerted “whole‐anion exchange” process in which an intact polybromide must first coordinate and then depart from the opposite side. Therefore, if a Grotthuss‐type transport mechanism were operative for polybromides, it would most plausibly proceed via the exchange of neutral Br_2_. However, analogous computational studies on polyiodides provide a theoretical basis to the contrary: the energy barrier for the exchange of neutral I_2_ is reported to be significantly higher (∼20 kcal mol^−1^) compared to that for I^−^ hopping (1–2 kcal mol^−1^) [35]. Based on these findings, it is reasonable to infer that a similarly high kinetic barrier likely exists for the exchange of neutral Br_2_ units within the PBIL.

Nevertheless, in the absence of direct computational or spectroscopic evidence, and since conducting such in‐depth studies lies beyond the scope of this work, we cautiously propose that polybromides are more likely transported by a vehicle‐type mechanism, suggesting that the Grotthuss mechanism is operative only for bromide ions. It should also be noted that this interpretation is based solely on impedance analysis, and thus should be regarded as suggestive rather than definitive. COMSOL simulations were performed using the obtained parameters to further validate the observed impedance asymmetry, with details of the simulations and discussion of the results provided in Supporting Information: Note S7.

### 2.2 Impact of Mass Transport Asymmetry on Dual‐Plating Zinc–Bromine Battery Performance

**FIGURE 4:**
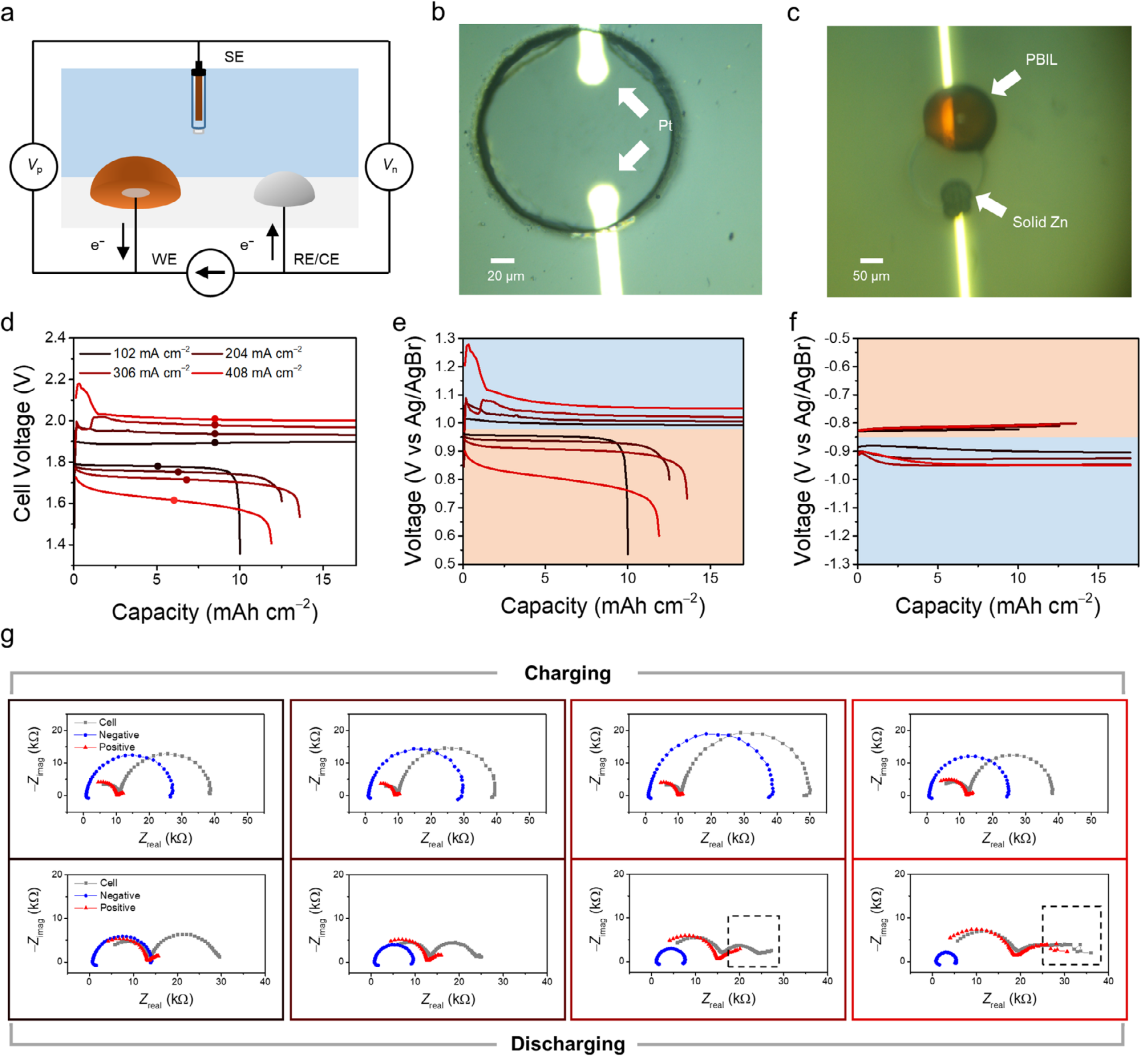
(a) Schematic illustration of voltage response obtained during a galvanostatic experiment using a custom‐built four‐electrode potentio‐/galvanostat, where *V*_p_ and *V*_n_ denote the positive and negative electrode potentials relative to the reference electrode, respectively. Optical microscope images of the fabricated micro ZBB, taken (b) before and (c) during charging. (d) Cell voltage profile during galvanostatic charge/discharge of a dual‐plating ZBB at varying current densities. Colored dots correspond to the midpoint of each charge/discharge phase. Voltage profiles of the (e) positive and (f) negative electrode, obtained separately by dual‐reference FTEIS, with the blue region denoting charging and the orange region denoting discharging. (g) Impedance of the positive electrode, negative electrode, and total cell measured at the midpoint of each charge (upper row) and discharge (lower row) phase in (d). The border color of each Nyquist plot matches the current density in (d). The dashed box highlights the emergence of a mass transport tail in the positive electrode and total cell impedance. The Nyquists are shown for frequencies ranging from 400 kHz to 10 Hz.

Building on the previous findings, we further investigated the impact of diffusion asymmetry on dual‐plating ZBB performance. A dual Pt UME chip was fabricated by photolithography (Figures S3 and S4) and used as a micro dual‐plating ZBB with an aqueous electrolyte of 0.5 M ZnSO_4_+0.25 M MEPBr. Moreover, we employed a custom‐built four‐electrode potentio‐/galvanostat, which, unlike commercial potentiostats, incorporates an extra voltage sensing terminal. This setup enables the separate measurement of positive and negative electrode potentials relative to an Ag/AgBr reference electrode, allowing for the isolation of the impedance responses of the positive and negative electrode during galvanostatic charge/discharge (Figure 4a). We refer to this technique as dual‐reference FTEIS. The micro ZBB cell was galvanostatically charged and discharged at currents ranging from 1 to 4 µA, corresponding to 102 to 408 mA cm^−2^, with the total charge fixed at 600 µC, corresponding to 17 mAh cm^−2^. The high conductivity of the PBIL phase enabled charging and discharging at exceptionally high current densities, far exceeding the typical range of 1–10 mA cm^−2^ for conventional battery cells [24]. In contrast, when tetrabutylammonium bromide (TBABr), a solid crystal‐forming BCA, was used instead of MEPBr, the ZBB operating 102 mA cm^−2^ failed to sustain discharge due to a sharp voltage drop (Figure S19), underscoring the importance of IL‐forming BCAs for fast cycling.

Optical microscope images of the micro ZBB before and after charging are shown in Figure 4b,c, while the voltage profiles are presented in Figure 4d and Figure S20. Charging the cell leads to the formation of a solid zinc phase on the negative electrode side and an orange‐colored PBIL phase on the positive electrode side (Figure 4c). The voltage profile of 102 mA cm^−2^ in Figure 4d indicates a working cell voltage of approximately 1.8 V, which aligns with the typical operating voltage of a ZBB. Higher overpotentials were observed at higher current densities during both charging and discharging, primarily thought of due to the increased ohmic drop. Notably, unlike the relatively stable charging curves, which show minimal overpotential buildup, the discharge curves reveal a continuous increase in overpotential throughout discharge, which becomes markedly intensified at higher current densities. Additionally, plotting the midpoint potentials of each charge and discharge step (Figure S21) reveals a steep, nonlinear increase in overpotential during discharge—behavior that would be linear if it originated solely from an ohmic contribution.

The separate positive and negative electrode potentials were monitored (Figure 4e,f), in order to find the origin of the unstable discharging curve. During charging, both electrodes exhibited stable voltage responses, with the overpotential increasing linearly with current density. The negative electrode voltage at 408 mA cm^−2^, appearing similar to that at 306 mA cm^−2^, can be explained by the order of galvanostatic cycling (306, 204, 102, and then 408 mA cm^−2^), reflecting a “memory effect” in which residual zinc remains after each cycle. Optical microscopy confirmed that the PBIL on the positive electrode was stripped more rapidly than the solid zinc on the negative electrode, leaving zinc deposits after each cycle that increase the active electrode surface area, thereby lowering the negative electrode impedance and overpotential (Figure S22).

When examining the potential profiles during discharge, two key features were observed: (1) the positive electrode potential exhibited instability, with progressive overpotential buildup and a nonlinear increase in overpotential, whereas (2) the negative electrode potential remained stable, showing virtually no additional ohmic drop. These separated potential profiles indicate that the unstable discharge behavior originates from the positive electrode. Given that the charge transfer process at the positive electrode interface is symmetric (Figure 3d), this suggests that charge transfer kinetics are likely not the primary factor driving the observed performance asymmetry between charging and discharging. To investigate the cause of this anomalous response, impedance spectra were further monitored during galvanostatic cycling using dual‐reference FTEIS.

The decoupled negative and positive electrode impedance, along with the full‐cell impedance for each current density at the midpoint of the charge/discharge phase, are shown in Figure 4g. Overall, the negative electrode‐side Nyquist plots display a single semicircle, which is assigned to the charge transfer RC process, as the characteristic frequency was found to be several hundred Hz—too high to correspond to the mass transport process of ions in the aqueous phase (Figure S23). Additionally, the negative electrode‐side impedance was successfully fitted using the equivalent circuit model *R*_s_(*R*_p_ *C*_dl_), which would not have been appropriate if mass transport were the dominant process (Table S3). The positive electrode‐side Nyquist plots exhibited a similar profile to previous results (Figures 2 and 3), showing a high‐frequency charge transfer arc followed by a low‐frequency mass transport tail.

During the charge phase (upper row of Figure 4g), the positive electrode Nyquist plots (red)—associated with PBIL formation—contribute minimally, whereas the negative electrode Nyquist plots (blue)—associated with zinc deposition—dominate the total cell response (gray) at all current densities. As noted above, the size of the negative electrode Nyquist plots decreases with the cycling sequence, reflecting the presence of residual zinc. The positive electrode Nyquist plots exhibit only a weak mass transport impedance tail, even at high current densities; both the charge transfer resistance and mass transport contribution remain largely unaffected by increasing current density. Although the concentration of ZnSO_4_ (0.5 M) is twice that of MEPBr (0.25 M), the cell impedance is still governed by the negative electrode side. This behavior can be explained by the extremely high Br^−^ concentration and diffusion coefficient within the PBIL, which facilitates rapid Br^−^ shuttling to the electrode and thereby markedly reduces polarization resistance. This also explains the significant difference in deposition current observed in Figure 1b.

In contrast, during the discharge phase, presented in the lower row of Figure 4g, the mass transport tail in the Nyquist plot of the positive electrode (red) gradually emerges as the current density increases. Furthermore, the high‐frequency arc, which corresponds to the heterogeneous charge transfer process (*R*_p_), increases at higher current densities. As noted above, *R*_p_ is expected to decrease with increasing overpotential when the surface concentration remains essentially unchanged (charge transfer limited; Figure 3d). However, the observed increase in *R*_p_ suggests that the concentration of Br_2n+1_^−^ near the electrode surface is being depleted, likely due to the relatively sluggish diffusion of Br_2n+1_^−^. Meanwhile, the negative electrode impedance, associated with the zinc dissolution reaction, decreases with increasing current density, which can be attributed to the higher overpotential accelerating the dissolution process. The total cell impedance gradually reflects the increasing impedance of the positive electrode side, also displaying an evident tail similar to that of the positive electrode impedance. This dominance of the positive electrode is explicitly quantified in Figure S24, which shows that the total cell impedance eventually follows the increasing trend of the positive electrode, overriding the decreasing trend of the negative electrode. Despite the term “dual‐plating” ZBB, PBIL dissolution does not proceed like a metal dissolution process, where dissolution takes place simultaneously across the entire deposited metal surface in electrical contact; instead, it relies on the supply of Br_2n+1_^−^ from the PBIL phase to the electrode, a process that is relatively sluggish compared to Br^−^ transport.

Further examining the voltage profile in Figure 4d, an unusual trend can be observed: the discharge capacity does not exhibit an inverse correlation with current density. Generally, regardless of the battery type, discharge capacity decreases with increasing current density due to enhanced polarization effects, particularly increased internal diffusion limitations within the active material [19, 26, 53, 54, 55]. However, when plotting discharge capacity versus current density (Figure S25), a quadratic dependency is observed. This distinct behavior can be explained by two different mechanisms. In the high current density region, the capacity decay is primarily driven by the sluggish mass transport of Br_2n+1_^−^ at the positive electrode. As shown in Figure S24, the positive electrode impedance exhibits a monotonically increasing trend with current density. This increased resistance induces severe concentration polarization, leading to a premature voltage cutoff and incomplete utilization of the active material. Conversely, in the low current density region, the capacity fading can be attributed to the time‐dependent diffusional loss. Since the capturing efficiency of MEP^+^ is not 100%, polybromides are not perfectly confined within the PBIL phase but gradually dissolve into the aqueous phase. The prolonged operation time at lower currents significantly expands the time window for these species to diffuse away from the electrode interface into the bulk electrolyte, resulting in the loss of redox‐active polybromide. Consequently, the discharge capacity maximizes at moderate current densities, representing a balance between incomplete utilization caused by mass‐transport limitations and active material loss caused by time‐dependent diffusion.

To examine the impact of asymmetric transport rates of bromine species on macro‐scale dual‐plating ZBBs, we used a home‐made cell with an active area of 4 cm^2^ (Figure S26). For ZBBs, operation at high current density is beneficial for suppressing Br_2n+1_^−^ crossover and improving cell efficiency. Tetraethylammonium bromide (TEABr), which tends to form a solid crystal phase rather than an IL phase, is commonly used in dual‐plating ZBBs. Stable charging and discharging can be achieved under low current density conditions (a few mA cm^−2^) [56, 57]. However, at high current densities (tens of mA cm^−2^), discharge becomes impossible, as shown in Figure S27. Specifically, charging at 10 mA cm^−2^ to an areal capacity of 50 mAh cm^−2^ leads to severe overpotential buildup, which is due to the TEABr_2n+1_ complex clogging the positive electrode (Figure S27b).

We also evaluated a cell employing TBABr, another BCA known to form solid polybromide complexes on the positive electrode surface (Figure S28) [26]. Although cycling was possible at current densities exceeding 10 mA cm^−2^—likely because the cell was operated at a reduced BCA concentration (0.1 M) due to solubility limits and a lower charging capacity (20 mAh cm^−2^) compared to the TEABr cell (0.3 M, 50 mAh cm^−2^, Figure S27), thereby significantly mitigating the extent of electrode clogging—the system exhibited severe charging overpotentials. Long‐term cycling at 10 and 20 mA cm^−2^ resulted in a severe aggravation of charging overpotentials and a subsequent loss of discharge capacity, attributed to the clogging of the positive electrode by solid polybromide salts (Figure S29).

**FIGURE 5:**
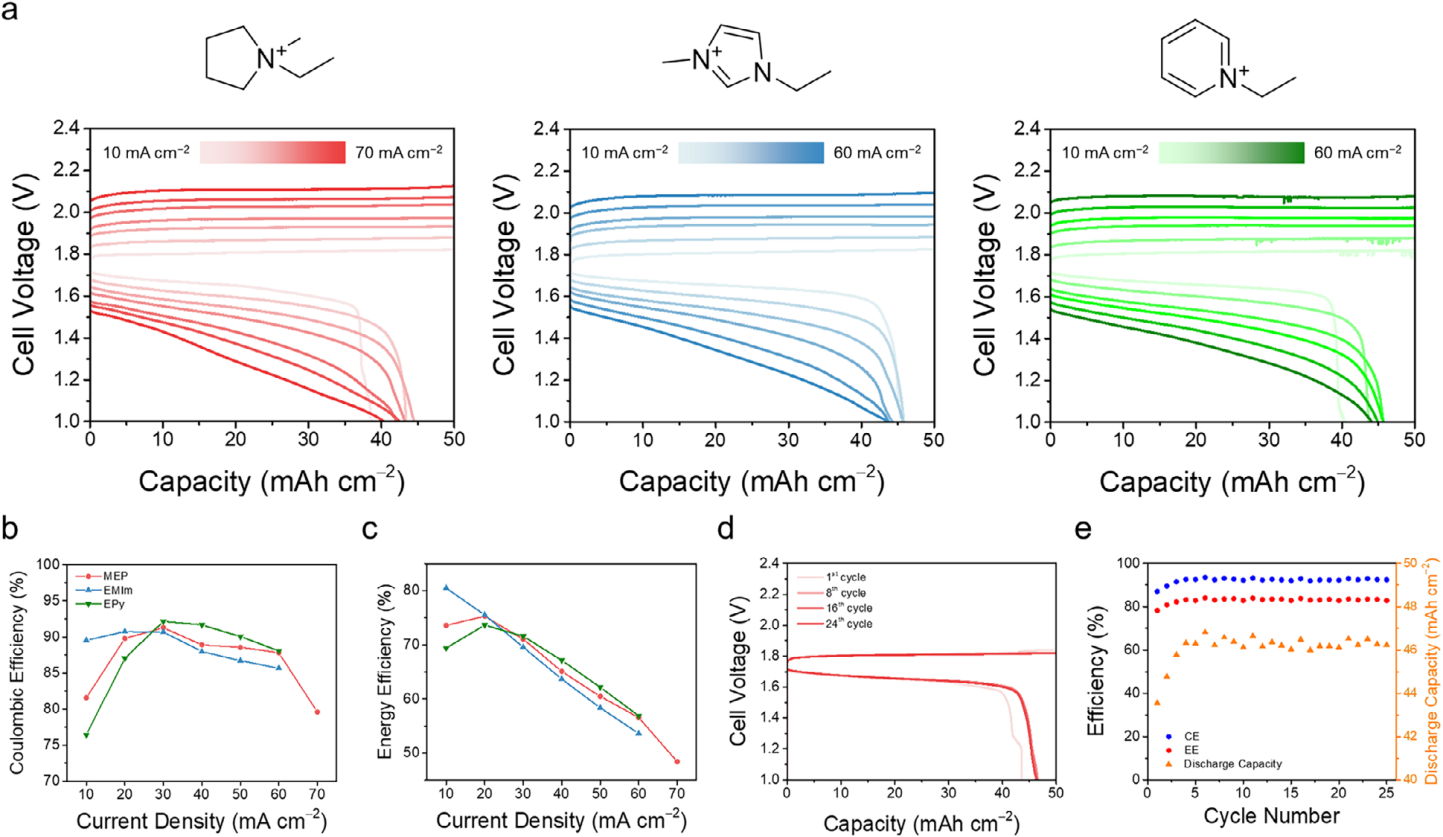
(a) Galvanostatic charge/discharge voltage profiles and corresponding, (b) coulombic efficiency (CE), and (c) energy efficiency (EE) of dual‐plating ZBBs employing three different IL‐forming BCAs: MEPBr, EMImBr, and EPyBr. The electrolytes consisted of 2.8 M ZnBr_2_ with 0.3 M of each respective BCA. The cells were operated at various current densities with a fixed areal charging capacity of 50 mAh cm^−2^. The reported efficiency values were derived by averaging three consecutive cycles at each current density. (d) Voltage profiles and (e) cycling stability metrics (CE, EE, and discharge capacity) of the MEPBr‐based macro‐scale ZBB under long‐term operation at 10 mA cm^−2^ for approximately 10 days.

In contrast, when evaluating various IL‐forming BCAs—including MEPBr, 1‐ethyl‐3‐methylimidazolium bromide (EMImBr), and 1‐ethylpyridinium bromide (EPyBr)—reversible cycling was feasible at high current densities of 60–70 mA cm^−2^ (Figure 5a). However, a distinct asymmetry was observed in the voltage profiles. While charging potentials remained relatively stable, the discharge process exhibited a systematic and significant increase in overpotential, particularly as the current density increased. While this phenomenon has been frequently reported in previous studies on dual‐plating zinc–halogen batteries, it has remained without a clear mechanistic explanation [37, 38]. Our findings suggest that this characteristic behavior can be attributed to the asymmetric transport rates of halide and polyhalide species within the IL phase. Given the fundamental similarities in polyhalide chemistry, we anticipate that this transport asymmetry is a universal characteristic governing the performance of other halogen‐based conversion batteries, such as dual plating zinc–iodine systems.

Notably, the quadratic dependency of Coulombic efficiency (CE) on current density was also observed in macro‐scale ZBBs across the tested BCAs (Figure 5b). Despite the decreasing voltage efficiency (VE) with increasing current density (Figure S30), the energy efficiency (EE) at 20 mA cm^−2^ was higher than at 10 mA cm^−2^ for MEPBr and EPyBr (Figure 5c). This highlights that a moderate discharge rate can be counterintuitively beneficial for certain IL‐ZBBs. As explained earlier, this behavior stems from two competing physical origins: at higher current densities, the shortened operation time suppresses Br_2n+1_^−^ crossover, enhancing capacity retention. However, at excessively high current densities, the sluggish Br_2n+1_^−^ transport causes severe polarization and premature voltage cutoff, which leads to an apparent decrease in CE due to incomplete discharge. These results suggest that an asymmetric operation protocol is essential for high‐performance dual‐plating ZBBs: high‐rate charging to minimize self‐discharge losses by leveraging fast Br^−^ transport, coupled with lower‐rate discharging to mitigate overpotentials arising from sluggish Br_2n+1_^−^ transport. However, the optimal current densities are not fixed; they depend on the cell's areal capacity and target energy efficiency, as shown in Figure S31. Additionally, implementing pulsed discharge or resting steps during high‐rate discharging could help mitigate surface depletion of Br_2n+1_^−^.

We further investigated the long‐term cycling stability of the MEPBr‐based ZBB to test the system's chemical robustness. When cycled at 10 mA cm^−2^ for over 10 days (Figure 5d,e), the cell exhibited highly reversible behavior over 25 cycles with a high areal capacity of 50 mAh cm^−2^. This stability highlights a fundamental difference between dual‐plating systems and conventional batteries with fixed active materials. In conventional systems, low coulombic efficiency often implies the irreversible consumption of the limited active material inventory, which accumulates over cycles to cause capacity fading. In contrast, dual‐plating ZBBs do not rely on a fixed solid host but operate by plating active species from an abundant electrolyte reservoir. Therefore, efficiency losses do not accumulate as permanent capacity loss, allowing the cell to maintain consistent capacity over repeated cycles. Furthermore, these results underscore the superior cycling performance of IL‐based ZBBs; the MEPBr system enabled stable long‐term operation under conditions where the TBABr system failed—exhibiting significant charging overpotential buildup and capacity loss at the same current density, even with a much lower areal capacity of 20 mAh cm^−2^ (Figure S29a).

Collectively, these results identify the sluggish mass transport of polybromides as the primary mechanistic origin of the rate‐dependent discharge anomalies observed in both micro‐ and macro‐scale ZBBs. Furthermore, these findings offer potential strategies for designing next‐generation BCAs to mitigate transport asymmetry. First, reducing the viscosity of the PBIL phase appears to be critical. Since the bulky polybromide ions are likely transported via a vehicle mechanism, designing cations that inherently lower the viscosity of the resulting ionic liquid (e.g., by increasing cation asymmetry) is expected to facilitate their mass transport. Second, tuning the cation‐anion interaction could be advantageous. Specifically, electrostatic interactions could be modulated to mitigate excessive binding that hinders diffusion—for instance, by introducing partially negative functional groups—while preserving the high local Br_2n+1_^−^ concentration required to sustain efficient Grotthuss transport of Br^−^.

## 3 Conclusion

This study underscores the critical role of diffusion asymmetry in the performance of dual‐plating ZBBs employing IL‐forming BCAs. Through a combination of electrochemical techniques, we demonstrated that the PBIL phase enhances Br^−^ transport during its formation (charging phase) while exhibiting significant mass‐transport limitations during its dissolution (discharging phase). FTEIS analysis suggests a pronounced contrast in the transport rates of Br^−^ and Br_2n+1_^−^ within the PBIL phase. COMSOL simulations further clarified the observed impedance asymmetry, demonstrating that during PBIL formation, the mass‐transport demand is sustained by the rapid diffusion of Br^−^, whereas during PBIL dissolution, it is met primarily by the abundance of Br_2n+1_^−^.

*Operando* dual‐reference FTEIS analysis using a micro‐scale dual‐plating ZBB revealed that during charging, rapid Br^−^ transport within the PBIL phase minimizes the positive electrode impedance, while the comparatively slow transport of Br_2n+1_^−^ leads to increased positive electrode impedance and mass‐transport limitations during discharging. The instability of the discharge voltage profile was directly correlated with the impedance trends of the positive electrode. Similar phenomena were observed in macro‐scale ZBBs across various IL‐forming BCAs, highlighting the impact of mass‐transport asymmetry on practical cell performance. Overall, this work advances the mechanistic understanding of the mass‐transport dynamics within the PBIL phase of dual‐plating ZBBs and provides insights into enhancing their efficiency under high–current density operation. Specifically, we propose that future BCA designs should focus on reducing PBIL viscosity and modulating cation‐anion interactions to mitigate these intrinsic transport limitations. Beyond mechanistic understanding, this study establishes FTEIS and dual‐reference FTEIS as powerful operando platforms capable of revealing the origins of anomalous battery behavior and guiding the rational design of next‐generation energy storage systems.

## 4 Experimental Section/Methods

The preparation of chemicals; methods for FTEIS measurements and impedance data fitting; electrochemical and optical measurements; large‐scale synthesis of MEPBr_2n+1_ ionic liquid; fabrication of micro zinc–bromine batteries; and galvanostatic operation at high areal capacities are described in the Supporting Information 58, 59, 60, 61, 62.

## Funding

This research Was Supported By the National Research Foundation of Korea (NRF) Grant Funded By the Korea government(MSIT) (No. RS‐2021‐NR060082 and RS‐2022‐NR070547).

## Conflicts of Interest

The authors declare no conflicts of interest.

## Supporting information




**Supporting File**: advs74264‐sup‐0001‐SuppMat.pdf.

## Data Availability

The data that support the findings of this study are available in the supplementary material of this article.

## References

[1] J. F. Parker , C. N. Chervin , I. R. Pala , et al., “Rechargeable Nickel–3D Zinc Batteries: An Energy‐Dense, Safer Alternative To Lithium‐Ion,” Science 356 (2017): 415–418, 10.1126/science.aak9991. 28450638

[2] F. Wang , O. Borodin , T. Gao , et al., “Highly Reversible Zinc Metal Anode for Aqueous Batteries,” Nature Materials 17 (2018): 543–549, 10.1038/s41563-018-0063-z. 29662160

[3] Z. Zheng , X. Zhong , Q. Zhang , et al., “An Extended Substrate Screening Strategy Enabling a Low Lattice Mismatch for Highly Reversible Zinc Anodes,” Nature Communications 15 (2024): 753, 10.1038/s41467-024-44893-0. PMC10810881 38272872

[4] H. Kim , K. M. Kim , J. Ryu , et al., “Triiodide‐in‐iodine Networks Stabilized by Quaternary Ammonium Cations as Accelerants for Electrode Kinetics of Iodide Oxidation in Aqueous media,” ACS Applied Materials & Interfaces 14 (2022): 12168–12179, 10.1021/acsami.1c21429. 35254047

[5] J.‐H. Lee , Y. Byun , G. H. Jeong , et al., “High‐Energy Efficiency Membraneless Flowless Zn–Br Battery: Utilizing the Electrochemical–Chemical Growth of Polybromides,” Advanced Materials 31 (2019): 1904524, 10.1002/adma.201904524. 31650656

[6] Z. Wei , Z. Huang , G. Liang , et al., “Starch‐mediated Colloidal Chemistry for Highly Reversible Zinc‐based Polyiodide Redox Flow Batteries,” Nature Communications 15 (2024): 3841, 10.1038/s41467-024-48263-8. PMC11076626 38714710

[7] C. Wang , G. Gao , Y. Su , et al., “High‐Voltage And Dendrite‐Free Zinc‐Iodine Flow Battery,” Nature Communications 15 (2024): 6234, 10.1038/s41467-024-50543-2. PMC11266666 39043688

[8] A. Mahmood , Z. Zheng , and Y. Chen , “Zinc–Bromine Batteries: Challenges, Prospective Solutions, and Future,” Advanced Science 11 (2024): 2305561, 10.1002/advs.202305561. 37988707 PMC10797452

[9] L. She , H. Cheng , Z. Yuan , et al., “Rechargeable Aqueous Zinc–halogen Batteries: Fundamental Mechanisms, Research Issues, and Future Perspectives,” Advanced Science 11 (2024): 2305061. 37939285 10.1002/advs.202305061 PMC10953720

[10] L. Tang , H. Peng , J. Kang , et al., “Zn‐based Batteries for Sustainable Energy Storage: Strategies and Mechanisms,” Chemical Society Reviews 53 (2024): 4877–4925, 10.1039/D3CS00295K. 38595056

[11] Z. Yan , Q.‐H. Yang , and C. Yang , “Elemental Halogen Cathodes for Aqueous Zinc Batteries: Mechanisms, Challenges and Strategies,” Journal of Materials Chemistry A 12 (2024): 24746–24760, 10.1039/D4TA05108D.

[12] Y. Liu , J. Meng , F. Yu , et al., “Synergistic Electrolyte Design for High‐Performance Static Zinc–Bromine Batteries,” ACS Energy Letters 10 (2025): 5809–5824, 10.1021/acsenergylett.5c02463.

[13] J. Meng , G. Zhang , L. Pang , et al., “Zinc–bromine Batteries Revisited: Unlocking Liquid‐Phase Redox Chemistry For Next‐Generation Energy Storage,” Energy & Environmental Science 18 (2025): 9031–9053, 10.1039/D5EE02219C.

[14] L. Li , J. Yao , J. Meng , et al., “Make Past Serve Present: A Novel Aqueous Lead–Bromine Battery With High Energy Density,” Materials Research Bulletin 184 (2025): 113250, 10.1016/j.materresbull.2024.113250.

[15] Y. Wang , Z. Yi , W. Luo , et al., “Suppression of Self‐discharge in a Non‐flowing Bromine Battery via in Situ Generation of Countercharged Groups,” Cell Reports Physical Science 2 (2021): 100620, 10.1016/j.xcrp.2021.100620.

[16] Q. Wang , Q. Dou , G. Deng , et al., “A Hybrid‐aqueous Biphasic Electrolyte for Suppressed Shuttle Effects and Self‐discharge of Zinc Bromide Batteries,” Journal of Materials Chemistry A 12 (2024): 15658–15665, 10.1039/D4TA01798F.

[17] H. Wu , J. Hao , S. Zhang , et al., “Aqueous Zinc–Iodine Pouch Cells With Long Cycling Life and Low Self‐Discharge,” Journal of the American Chemical Society 146 (2024): 16601–16608, 10.1021/jacs.4c03518. 38840442

[18] X. Li , T. Li , P. Xu , C. Xie , Y. Zhang , and X. Li , “A Complexing Agent to Enable a Wide‐Temperature Range Bromine‐Based Flow Battery for Stationary Energy Storage,” Advanced Functional Materials 31 (2021): 2100133, 10.1002/adfm.202100133.

[19] M. Zhao , T. Cheng , T. Li , R. Bi , Y. Yin , and X. Li , “A Choline‐Based Antifreezing Complexing Agent With Selective Compatibility for Zn–Br_2_ Flow Batteries,” Small 20 (2024): 2307627, 10.1002/smll.202307627. 38063849

[20] C. Wang , Q. Xie , G. Wang , et al., “Visualizing and Understanding the Ionic Liquid‐Mediated Polybromide Electrochemistry for Aqueous Zinc‐Bromine Redox Batteries,” Nano Letters 24 (2024): 13796–13804, 10.1021/acs.nanolett.4c04167. 39401413

[21] S. Ito , M. Sugimasa , Y. Toshimitsu , A. Orita , M. Kitagawa , and M. Sakai , “Formation of a Hydrophobic Polyiodide Complex During Cathodic Oxidation of Iodide in the Presence of Propylene Carbonate in Aqueous Solutions, and Its Application to a Zinc/Iodine Redox Flow Battery,” Electrochimica Acta 319 (2019): 164–174, 10.1016/j.electacta.2019.06.150.

[22] J. Yang , Y. Song , Q. Liu , and A. Tang , “High‐Capacity Zinc–Iodine Flow Batteries Enabled By A Polymer–Polyiodide Complex Cathode,” Journal of Materials Chemistry A 9 (2021): 16093–16098, 10.1039/D1TA03905A.

[23] Y. Yin , Z. Yuan , and X. Li , “Rechargeable Aqueous Zinc–Bromine Batteries: An Overview And Future Perspectives,” Physical Chemistry Chemical Physics 23 (2021): 26070–26084, 10.1039/D1CP03987C. 34787128

[24] H. Zhao , D. Yin , Y. Qin , et al., “Highly Electrically Conductive Polyiodide Ionic Liquid Cathode for High‐Capacity Dual‐Plating Zinc–Iodine Batteries,” Journal of the American Chemical Society 146 (2024): 6744–6752, 10.1021/jacs.3c12695. 38422617

[25] H. Yang , S. Lin , Y. Qu , et al., “An Ultra‐Low Self‐Discharge Aqueous|Organic Membraneless Battery With Minimized Br_2_ Cross‐Over,” Advanced Science 11 (2024): 2307780, 10.1002/advs.202307780. 38168899 PMC10870083

[26] C. Dai , L. Hu , X. Jin , et al., “Fast Constructing Polarity‐Switchable Zinc‐Bromine Microbatteries With High Areal Energy Density,” Science Advances 8 (2022): abo6688, 10.1126/sciadv.abo6688. PMC9278868 35857517

[27] J. J. Hong , L. Zhu , C. Chen , et al., “A Dual Plating Battery With the Iodine/[ZnI_x_(OH_2_)_4−x_]^2−x^ Cathode,” Angewandte Chemie International Edition 58 (2019): 15910–15915, 10.1002/anie.201909324. 31478325

[28] M. Kim , S. Park , and T. D. Chung , “Heterogeneous Electron Transfer Reorganization Energy at the Inner Helmholtz Plane in a Polybromide Redox‐active Ionic Liquid,” Chemical Science 13 (2022): 8821–8828, 10.1039/D2SC01410F. 35975145 PMC9350599

[29] S. Park , D. H. Han , J. G. Lee , and T. D. Chung , “Current Amplification and Ultrafast Charge Transport in a Single Microdroplet of Bromide/Polybromide‐based Ionic Liquid,” ACS Applied Energy Materials 3 (2020): 5285–5292, 10.1021/acsaem.0c00207.

[30] S. Park , H. Kim , J. Chae , and J. Chang , “Electrochemical Generation of Single Emulsion Droplets and in Situ Observation of Collisions on an Ultramicroelectrode,” The Journal of Physical Chemistry C 120 (2016): 3922–3928, 10.1021/acs.jpcc.5b12029.

[31] S. Shin , D. Jung , J. Chae , and J. Chang , “Stochastic Electrochemical Analysis of Electrochemically Generated Ethylpyridinium Polybromide Droplets: Evidence of Br^−^/Br_3_ ^−^/Br_2_ Electro‐oxidation in Quaternary Ammonium Polybromide,” Journal of Electroanalytical Chemistry 802 (2017): 123–130, 10.1016/j.jelechem.2017.08.021.

[32] S. Park , S. Shin , D. Jung , J. Chae , and J. Chang , “Understanding Br − Transfer Into Electrochemically Generated Discrete Quaternary Ammonium Polybromide Droplet on Pt Ultramicroelectrode,” Journal of Electroanalytical Chemistry 797 (2017): 97–106, 10.1016/j.jelechem.2017.05.014.

[33] S. Lee , S. Park , K. M. Kim , and J. Chang , “Semi‐quantitative Determination of Ion Transfers at an Interface Between Water and Quaternary Ammonium Polybromide Droplets Through Stochastic Electrochemical Analysis,” Electrochimica Acta 271 (2018): 454–463, 10.1016/j.electacta.2018.03.162.

[34] V. K. Thorsmølle , G. Rothenberger , D. Topgaard , et al., “Extraordinarily Efficient Conduction in a Redox‐Active Ionic Liquid,” Chemphyschem 12 (2011): 145–149, 10.1002/cphc.201000819. 21226195

[35] J. G. McDaniel and A. Yethiraj , “Grotthuss Transport of Iodide in EMIM/I_3_ Ionic Crystal,” The Journal of Physical Chemistry B 122 (2018): 250–257, 10.1021/acs.jpcb.7b09292. 29195043

[36] J. K. Wlodarczyk , N. Baltes , K. A. Friedrich , and J. O. Schumacher , “Mass Transport Limitations In Concentrated Aqueous Electrolyte Solutions: Theoretical and Experimental Study Of The Hydrogen–Bromine Flow Battery Electrolyte,” Electrochimica Acta 461 (2023): 142640, 10.1016/j.electacta.2023.142640.

[37] H. Li , B. Huang , M. Chuai , et al., “Dual‐Plating Aqueous Zn–Iodine Batteries Enabled Via Halogen‐Complexation Chemistry For Large‐Scale Energy Storage,” Energy & Environmental Science 18 (2025): 3160–3168, 10.1039/D5EE00027K.

[38] T. Xiao , J.‐L. Yang , B. Zhang , et al., “All‐Round Ionic Liquids for Shuttle‐Free Zinc‐Iodine Battery,” Angewandte Chemie International Edition 63 (2024): 202318470, 10.1002/anie.202318470. 38179860

[39] T. Breugelmans , J. Lataire , T. Muselle , E. Tourwé , R. Pintelon , and A. Hubin , “Odd Random Phase Multisine Electrochemical Impedance Spectroscopy to Quantify a Non‐stationary Behaviour: Theory and Validation by Calculating an Instantaneous Impedance Value,” Electrochimica Acta 76 (2012): 375–382, 10.1016/j.electacta.2012.05.051.

[40] K. Cysewska , L. F. Macía , P. Jasiński , and A. Hubin , “In‐Situ Odd Random Phase Electrochemical Impedance Spectroscopy Study On The Electropolymerization Of Pyrrole On Iron In The Presence Of Sodium Salicylate—The Influence Of The Monomer Concentration,” Electrochimica Acta 290 (2018): 520–532, 10.1016/j.electacta.2018.09.069.

[41] S. H. Han , J. Rho , S. Lee , et al., “In Situ Real‐time Monitoring of ITO Film Under a Chemical Etching Process Using Fourier Transform Electrochemical Impedance Spectroscopy,” Analytical Chemistry 92 (2020): 10504–10511, 10.1021/acs.analchem.0c01294. 32489093

[42] B.‐Y. Chang and S.‐M. Park , “Integrated Description of Electrode/Electrolyte Interfaces Based on Equivalent Circuits and Its Verification Using Impedance Measurements,” Analytical Chemistry 78 (2006): 1052–1060, 10.1021/ac051641l. 16478095

[43] K. M. Nam , D. H. Shin , N. Jung , et al., “Development of Galvanostatic Fourier Transform Electrochemical Impedance Spectroscopy,” Analytical Chemistry 85 (2013): 2246–2252, 10.1021/ac303108n. 23331177

[44] S. I. Kim and T. D. Chung , “In Situ Real‐time Dendritic Growth Determination of Electrodeposits on Ultramicroelectrodes,” Analytical Chemistry 96 (2024): 3096–3106. 10.1021/acs.analchem.3c05239 38341845

[45] Y. Choi , J. Hwang , K. M. Kim , et al., “Time Transient Electrochemical Monitoring of Tetraalkylammonium Polybromide Solid Particle Formation: Observation of Ionic Liquid‐to‐solid Transitions,” Analytical Chemistry 91 (2019): 5850–5857, 10.1021/acs.analchem.9b00190. 30942070

[46] J. Heinze , “Ultramicroelectrodes in Electrochemistry,” Angewandte Chemie International Edition in English 32 (1993): 1268–1288, 10.1002/anie.199312681.

[47] M. W. Verbrugge and B. J. Koch , “Microelectrode Investigation of Ultrahigh‐rate Lithium Deposition and Stripping,” Journal of Electroanalytical Chemistry 367 (1994): 123–129, 10.1016/0022-0728(93)03047-S.

[48] M. A. Faisal and J. E. Dick , “A Battery Researcher's Guide to Successful Ultramicroelectrode Fabrication for Fast‐scan Kinetic Analysis,” ACS Applied Energy Materials 7 (2024): 10326–10334, 10.1021/acsaem.4c01747.

[49] A. Rana , M. A. Faisal , K. Roy , J. H. Nguyen , S. Paul , and J. E. Dick , “How the Kinetic Balance Between Charge‐transfer and Mass‐transfer Influences Zinc Anode Stability: An Ultramicroelectrode Study,” Small Methods 8 (2024): 2401021. 10.1002/smtd.202401021 PMC11926490 39564707

[50] A. J. Bard , L. R. Faulkner , and H. S. White , Electrochemical Methods: Fundamentals and Applications (Wiley, 2022).

[51] Q.‐A. Huang and S.‐M. Park , “Unified Model for Transient Faradaic Impedance Spectroscopy: Theory and Prediction,” The Journal of Physical Chemistry C 116 (2012): 16939–16950, 10.1021/jp306140w.

[52] Y. Choi , C. Park , Y. Kang , J. T. Muya , D. P. Jang , and J. Chang , “Temporally Resolved Electrochemical Interrogation for Stochastic Collision Dynamics of Electrogenerated Single Polybromide Droplets,” Analytical Chemistry 93 (2021): 8336–8344, 10.1021/acs.analchem.1c01366. 34075746

[53] U.‐H. Kim , S.‐B. Lee , N.‐Y. Park , S. J. Kim , C. S. Yoon , and Y.‐K. Sun , “High‐energy‐density Li‐ion Battery Reaching Full Charge in 12 Min,” ACS Energy Letters 7 (2022): 3880–3888, 10.1021/acsenergylett.2c02032.

[54] M. Ryu , Y.‐K. Hong , S.‐Y. Lee , and J. H. Park , “Ultrahigh Loading Dry‐process for Solvent‐free Lithium‐ion Battery Electrode Fabrication,” Nature Communications 14 (2023): 1316, 10.1038/s41467-023-37009-7. PMC10006413 36899006

[55] Y. Zhang , W. Bao , E. Jeffs , et al., “Unveiling the Impacts of Charge/Discharge Rate on the Cycling Performance of Li‐metal Batteries,” ACS Energy Letters 10 (2025): 872–880, 10.1021/acsenergylett.4c03215.

[56] L. Gao , Z. Li , Y. Zou , et al., “A High‐performance Aqueous Zinc–bromine Static Battery,” iScience 23 (2020): 101348. 32711343 10.1016/j.isci.2020.101348 PMC7387827

[57] S. S. Mollick , T. Mandal , and S. Ramakrishnan , “A High Energy Density, Non‐flow Zinc Bromine Battery Enabled by a Solubility Product Optimization Strategy,” Journal of The Electrochemical Society 172 (2025): 010503, 10.1149/1945-7111/ad9fe2.

[58] A. C. Lazanas and M. I. Prodromidis , “Electrochemical Impedance Spectroscopy─A Tutorial,” ACS Measurement Science Au 3 (2023): 162–193, 10.1021/acsmeasuresciau.2c00070. 37360038 PMC10288619

[59] A. Lasia , Electrochemical Impedance Spectroscopy and Its Applications (Springer, 2002).

[60] X. Chen , M. A. Rickard , J. W. Hull , C. Zheng , A. Leugers , and P. Simoncic , “Raman Spectroscopic Investigation of Tetraethylammonium Polybromides,” Inorganic Chemistry 49 (2010): 8684–8689, 10.1021/ic100869r. 20812678

[61] M. E. Easton , A. J. Ward , T. Hudson , et al., “The Formation of High‐order Polybromides in a Room‐temperature Ionic Liquid: From Monoanions ([Br_5_]− to [Br_11_]−) to the isolation of [PC_16_H_36_]_2_[Br_24_] as Determined by van der Waals bonding radii,” Chemistry–A European Journal 21 (2015): 2961–2965. 25487061 10.1002/chem.201404505

[62] M. Kuttinger , J. K. Wlodarczyk , D. Daubner , P. Fischer , and J. Tubke , “High Energy Density Electrolytes for H_2_/Br_2_ Redox Flow Batteries, Their Polybromide Composition and Influence on Battery Cycling Limits,” RSC Advances 11 (2021): 5218–5229, 10.1039/D0RA10721B. 35424436 PMC8694680

