# Supplementary material for: Asymmetric Mass Transport in Polybromide Ionic Liquids and Its Impact on Dual‐Plating Zinc Bromine Batteries
Source: Adv Sci (Weinh). 2026 Feb 8;13(21):e22078. doi: 10.1002/advs.202522078 (PMC13073245; doi:10.1002/advs.202522078)
Supplement: Supplementary file 1 — Supporting File: advs74264‐sup‐0001‐SuppMat.pdf. [file ADVS-13-e22078-s001.pdf]

Supporting Information

Asymmetric Mass Transport in Polybromide Ionic Liquids and its Impact on Dual-Plating Zinc Bromine Batteries

Sung Il Kim,[A]† Kyungjae Shin,[B]† Heejung Kang,[A] Seok Hee Han,[C] Hee-Tak Kim,[B] and Taek Dong Chung,*[A,D]*

†Sung Il Kim and Kyungjae Shin contributed equally to this work.

Experimental Section

Chemicals

1-ethyl-1-methylpyrrolidinium bromide (MEPBr), tetrabutylammonium bromide (TBABr), tetraethylammonium bromide (TEABr), zinc sulfate monohydrate ($ZnSO_4 \cdot H_2O$), zinc bromide ($ZnBr_2$) were purchased from Sigma-Aldrich. 0.5 M H_2SO_4 was purchased from Samchun Chemicals. All reagents were used without further purification.

FTEIS Measurements and Impedance Data Fitting

A homemade potentiostat was utilized for monitoring the impedance of the electrochemical system in real-time. An NI USB-6366 multifunction I/O device (NI, U.S.A) was employed for both waveform generation and data acquisition. Using LabVIEW programming, an odd random phase multisine signal was produced and sent to the homemade potentiostat via the USB-6366. The excitation signal was created by incorporating selected odd overtones of the fundamental frequency (the inverse of the multisine signal length), and the signal's peak-to-peak voltage was scaled to 50 mV. More detailed description of the FTEIS measurement can be found elsewhere.[44] The impedance spectra were simultaneously fitted to a suitable equivalent circuit using complex nonlinear least squares with the Levenberg−Marquardt algorithm. Additionally, the accuracy of the Nyquist plots obtained during the electrochemical experiments was assessed using Lin-KK, a freeware tool for automated linear Kramers−Kronig testing (**Figure S1**). The real and imaginary residuals were found to be within a reasonable range of 1%.

For zinc-bromine battery experiments, a homemade potentiostat/galvanostat equipped with a reference electrode and a separate voltage sensing terminal was utilized. The default configuration was a two-electrode system, with the working electrode and reference/counter electrode shorted, to apply voltage or current between one Pt electrode and the other. An Ag/AgBr reference electrode was connected to the voltage sensing terminal to separately record the voltage at each interface, enabling the measurement of the electrochemical impedance of each electrode.

Electrochemical, Optical Measurements

Electrochemical measurements other than FTEIS were conducted with an Autolab PGSTAT302N instrument (Metrohm, Switzerland). For all three-electrode electrochemical experiments, a platinum (Pt) wire was used as the counter electrode, and an Ag/AgBr (3 M KBr) electrode served as the reference electrode. An optical microscope (Olympus BXFM) with a 20× objective was utilized for optical imaging. The Raman spectra were recorded using a custom-built Ramboss Micro-Raman system, also featuring a 20× objective lens. A 633 nm line from a He-Ne laser (LASOS Lasertechnik GmbH, Jena, Germany) served as the excitation source. For calibration, Raman bands of a silicon wafer at 520 cm^{-1} was used.

Large-Scale Synthesis of $MEPBr_{2n+1}$ Ionic Liquid

$MEPBr_{2n+1}$ was electrochemically synthesized in large scale using a 200 nm Pt deposited silicon wafer as a working electrode. The working electrode was immersed in a solution containing 0.5 M MEPBr and 0.5 M H_2SO_4 and a constant potential of 1.3 V versus the reference electrode was applied for 10,000 s. The synthesized $MEPBr_{2n+1}$ ionic liquid was collected and was kept in a 20 mL vial with the electrolyte solution used for synthesis (**Figure S2**). The ionic liquid phase was immiscible in water and was stable semi-permanently.

Fabrication of Micro Zinc Bromine Battery

A patterned Pt chip was fabricated using photolithographic techniques, as illustrated in **Figure S3**, which outlines the complete fabrication process of the patterned Pt electrode for the micro zinc-bromine battery. First, a 200 nm Pt-deposited glass wafer was prepared via e-beam deposition, followed by spin-coating with AZ5214 photoresist. Using the photomask shown in **Figures S4a** and **S4b**, the photoresist was patterned to serve as a protective layer. The exposed Pt was then dry-etched, leaving only the protected regions, after which the AZ5214 was removed using acetone. To isolate the circular region in **Figure S4b** for use as the electrode, SU-8 photoresist was applied to cover the remaining Pt, leaving the circular region uncovered as the electrode and an additional small area uncovered to enable electrical contact with the

electrode. Stereoscopic microscope images of the fabricated chip are shown in **Figures S4c** and **S4d**. The two Pt electrodes had surface areas of 9.58×10^{-10} m^2 and 7.54×10^{-10} m^2. For the calculation of current density during galvanostatic charge-discharge, the surface area of 9.58×10^{-10} m^2 was used, as this electrode served as the positive electrode, and C-rate is conventionally based on the theoretical capacity of the positive electrode. The Pt chip was reusable after washing off the MEPBr PBIL with acetone and removing the solid Zn by immersing it in a 0.5 M H_2SO_4 solution.

Galvanostatic operation at high areal capacity

A dual-plating ZBB designed for high areal capacity was fabricated using a home-made cell with a 4 cm^2 active area and a 2 cm thickness, accommodating 8 mL of electrolyte. Graphite felt (Ceramaterials) was used for both electrodes. To enhance hydrophilicity, the graphite felt was thermally oxidized at 520℃ for 9 hours, with a heating rate of 5℃ min^{-1}. A glass fiber filter paper (GF/F, Whatman) was used as the separator without further treatment. The rate variation test was performed using a battery cycler (WBCS3000, OneATech). The cells were discharged galvanostatically until the voltage reached 1 V vs. Zn/Zn^{2+}.

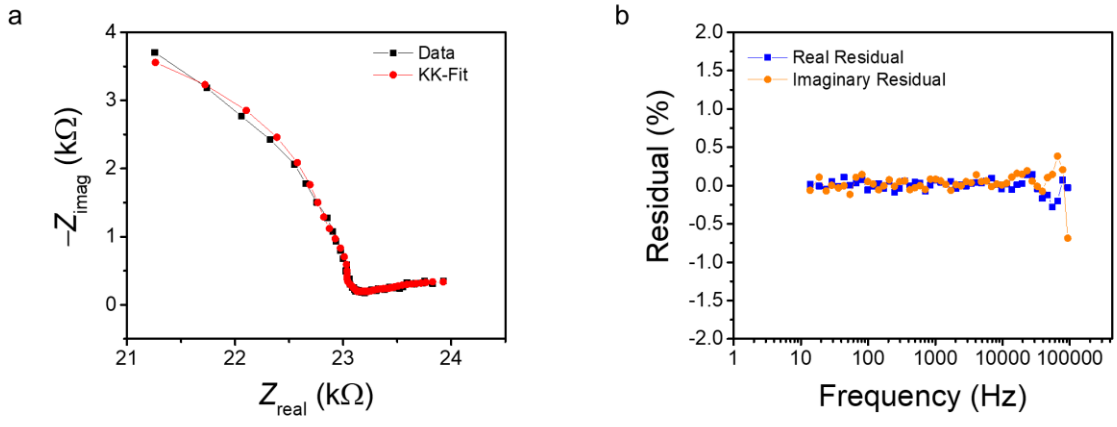

Figure S1. (a, b) Results of the linear Kramers-Kronig (Lin-KK) validity test. (a) Nyquist plot obtained with 5 µL of PBIL placed on a 10 µm Pt UME and immersed in 0.5 M H_2SO_4 (black), alongside its Kramers-Kronig fit (red). (b) Real and imaginary residuals from the Lin-KK test, shown in blue and orange, respectively.

a b

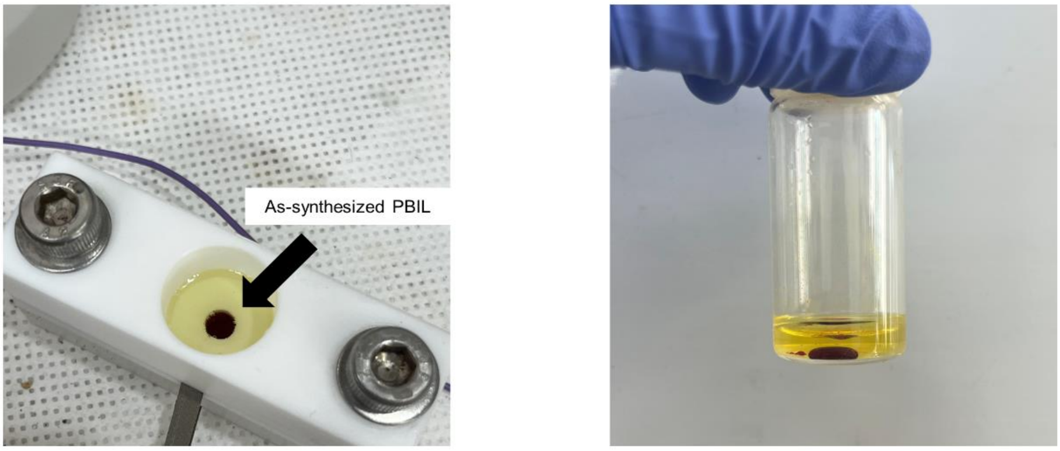

Figure S2. (a) Photograph of electrochemically synthesized PBIL immediately after synthesis. (b) Photograph of the same PBIL after being stored for 3 months in the electrolyte used for its synthesis.

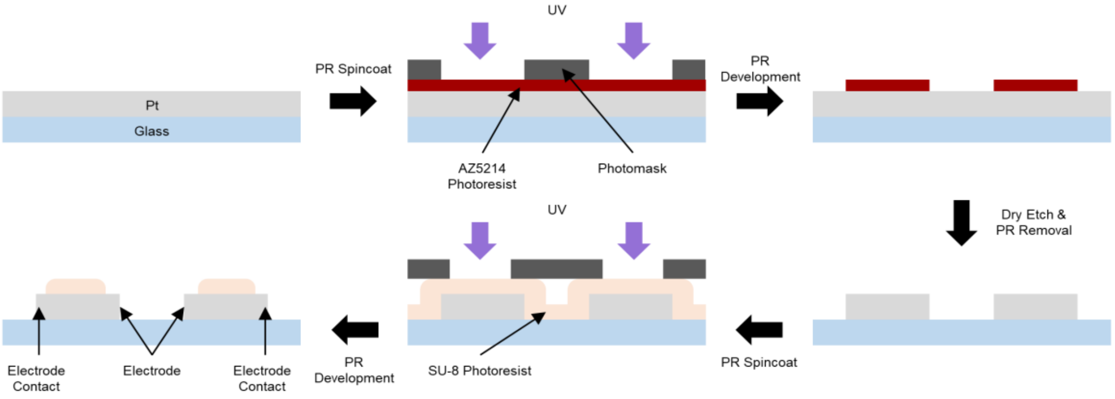

Figure S3. Schematic representation of the fabrication process for dual Pt UME chip designed for ZBB applications.

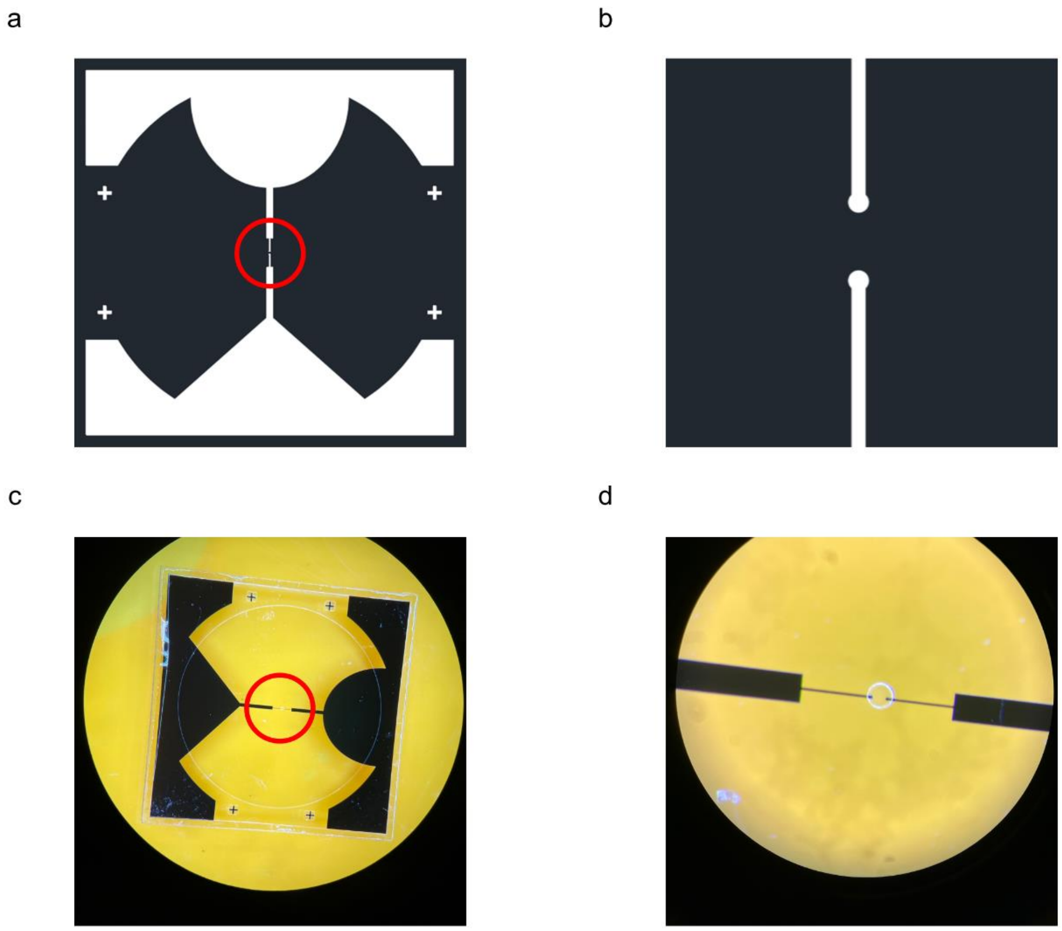

Figure S4. (a) Design of the photomask used for AZ5214 UV exposure. (b) Magnified view of the red-circled area in (a). (c) Stereoscopic microscope image of the fabricated dual Pt UME chip. (d) Magnified view of the red-circled area in (c).

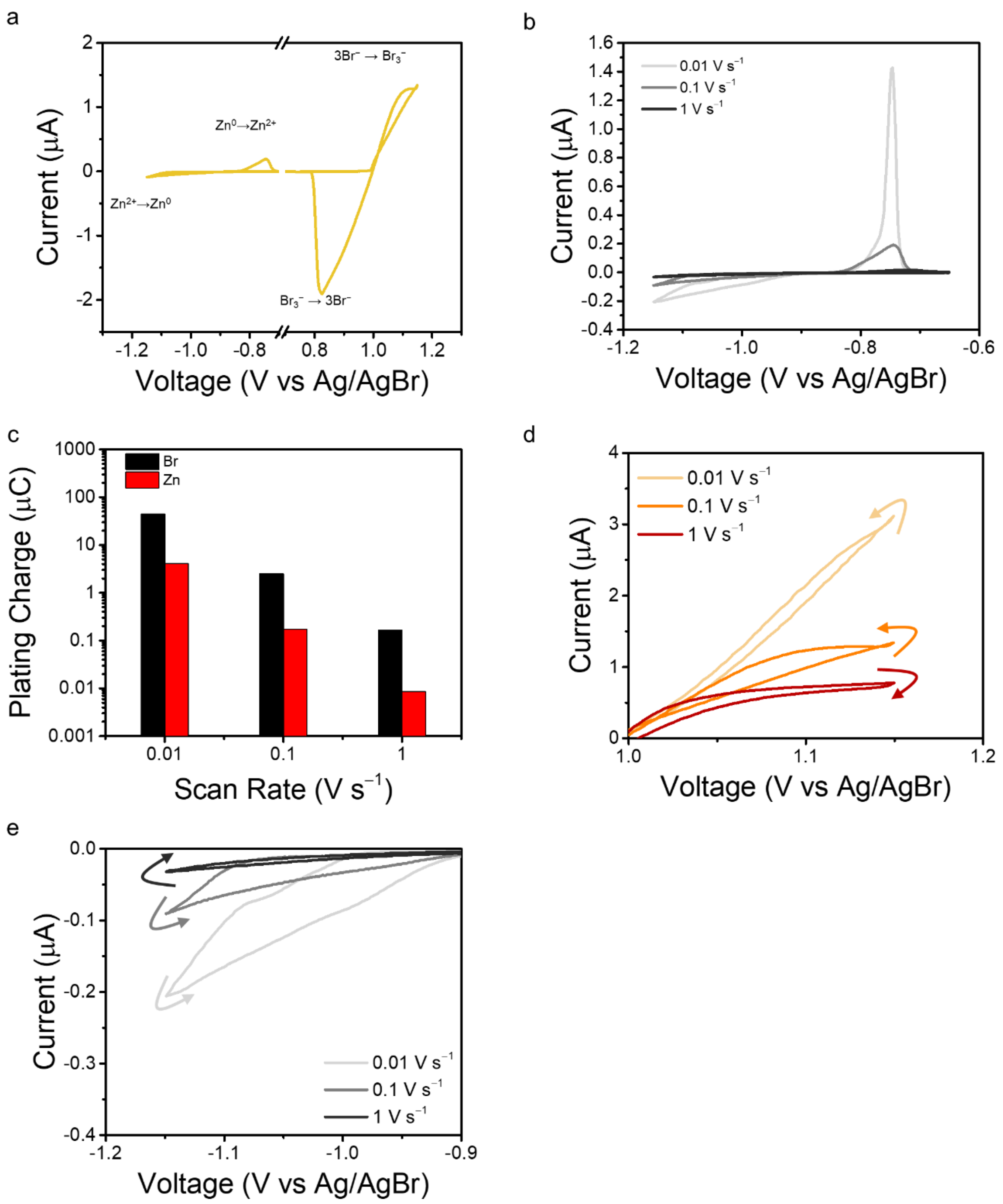

Figure S5. (a) Cyclic voltammograms recorded from a 10 μm Pt UME immersed in 0.5 M ZnSO4 + 0.25 M MEPBr with a scan rate of 0.1 V s^{-1}. (b) Non-amplified cyclic voltammogram corresponding to Figure 1c. (c) Charge passed during the formation step for PBIL and solid Zn at each scan rate, as depicted in the cyclic voltammograms in Figure 1. Magnified views of the (d) PBIL and (e) solid Zn plating potential regions in the cyclic voltammograms of Figure 1.

Note S1. Basics of Electrochemical Impedance Spectroscopy

Figure S6a schematically illustrates the physicochemical processes occurring under high- and low-frequency voltage perturbations during EIS measurements. At high-frequency voltage perturbations, redox species near the electrode surface are reduced or oxidized through heterogeneous electron transfer. Consequently, the current response at high frequency reflects the heterogeneous charge transfer kinetics, with smaller impedance values indicating faster charge transfer rates. In contrast, at low-frequency voltage perturbations, the longer duration of each perturbation cycle leads to extended reaction times, eventually resulting in the depletion of redox species near the electrode surface. To sustain current flow under these conditions, redox species must be transported from the bulk solution to the electrode surface. This mass transport process limits the overall current, indicating that the low-frequency impedance provides information about the mass transport properties of the redox species.

In general, impedance data obtained from a three-electrode electrochemical system involving a simple redox reaction ($O + ne^- \rightleftarrows R$) is commonly fitted to the Randles equivalent circuit. This circuit includes the solution resistance (R_s), double layer capacitance (C_{dl}, or Q_{dl} for non-ideal capacitive behavior), charge transfer resistance (R_{ct}, often denoted as R_p for polarization resistance), and a Warburg element (Z_W) representing mass transport.[58] **Figure S6b** illustrates a Randles circuit with a spherical-diffusion Warburg element, instead of semi-infinite linear-diffusion Warburg element, which is commonly used for macroelectrodes. The mathematical expression of the Warburg element varies with the electrode or cell geometry. When visualized in a Nyquist plot, the impedance typically exhibits a high-frequency semicircle corresponding to charge transfer processes, appearing in the low-impedance region, and a low-frequency region associated with mass transport, appearing in the high-impedance region.[58, 59]

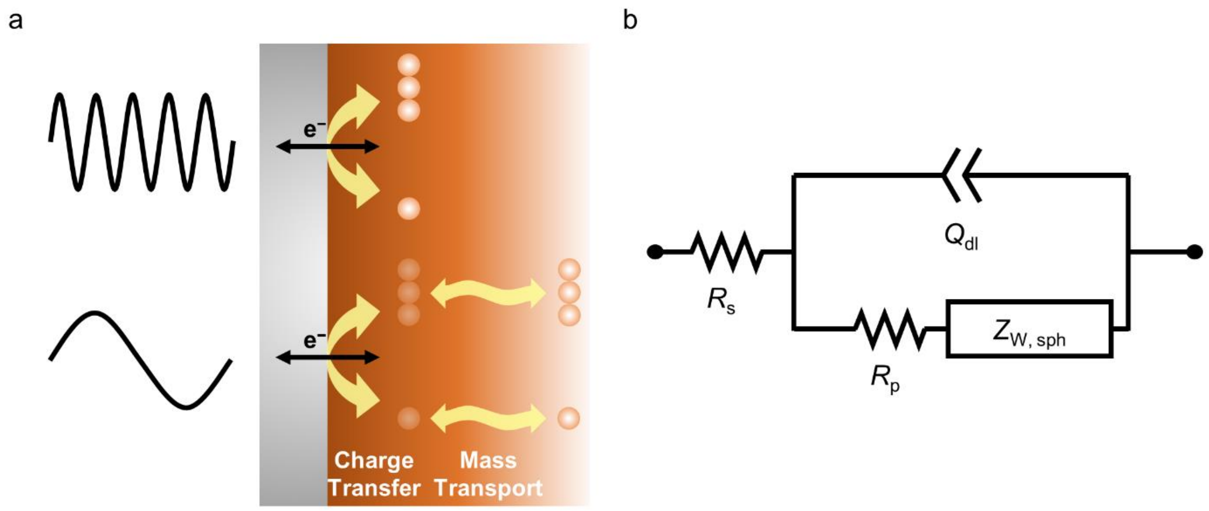

Figure S6. (a) Illustration of the physical processes analyzed via EIS, highlighting that charge transfer processes are probed at high frequencies while mass transport processes are probed at low frequencies. (b) Randles circuit used for impedance fitting throughout the study, containing solution resistance (R_s), double layer capacitance (Q_{dl}), charge transfer resistance (R_p), and a spherical diffusion Warburg element ($Z_{W,sph}$).

Note S2. Shift in Nyquist Plot upon Formation of PBIL Phase

As the PBIL phase forms on the electrode surface, the concentration of redox species becomes highly concentrated near the surface.[29] This can cause a decrease in charge transfer resistance and also the RC time constant. The reduction in the charge transfer time constant shifts the frequency range of the charge transfer process to a higher region, consequently shifting the frequency range of the mass transport process as well, as mass transport must occur following the completion of the charge transfer process. The shift in the characteristic frequency of the charge transfer process, which is the inverse of the charge transfer time constant (RC), is shown in **Figure S7**. This frequency corresponds to the peak point of the charge transfer arc in the Nyquist plot. The shift in shape of mass transport impedance can be explained by further looking at the diffusion impedance expression.

The spherical diffusion Warburg impedance is expressed as follows:[58, 59]

$$Z_{\mathrm{W,sph}} = \sum_{i=O,R} \frac{\sigma_i{}'}{\sqrt{j\omega} + \frac{\sqrt{D_i}}{r_0}}$$

where σ_i', D_i, r_0, j, and ω represent the Warburg coefficient, diffusion coefficient, electrode radius, imaginary unit, and angular frequency of the AC signal, respectively. The $\sqrt{j\omega}$ term corresponds to the contribution from linear diffusion, while the $\sqrt{D_i}/r_0$ term represents the spherical diffusion component. As the initiation frequency of mass transport shifts to a higher range due to PBIL formation, this results in an overall increase in $\sqrt{j\omega}$, enhancing the linear diffusion contribution. Since the linear and spherical diffusion terms compete in the denominator of $Z_{\mathrm{W,sph}}$, the increase in $\sqrt{j\omega}$ causes the spherical diffusion term $\sqrt{D_i}/r_0$ to become negligible. Consequently, the shape of the mass transport impedance transitions from a depressed arc—characteristic of spherical diffusion—to a more linear form, indicative of 1D linear diffusion.

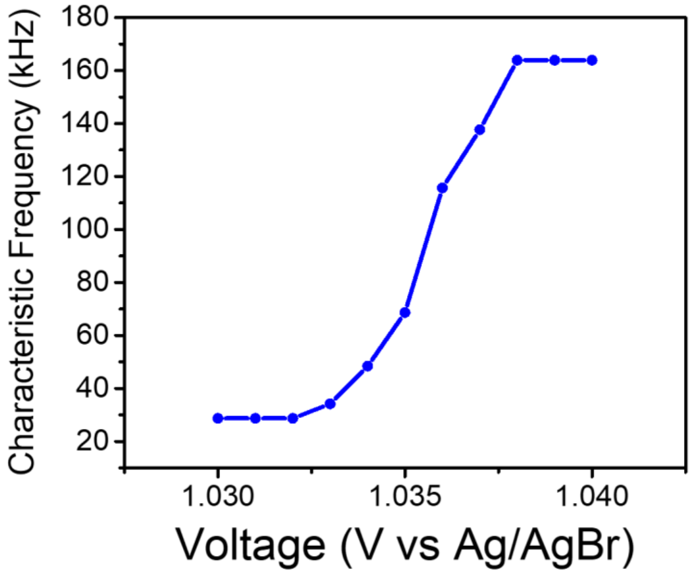

Figure S7. Transition of the characteristic frequency of the charge transfer process as a function of electrode voltage.

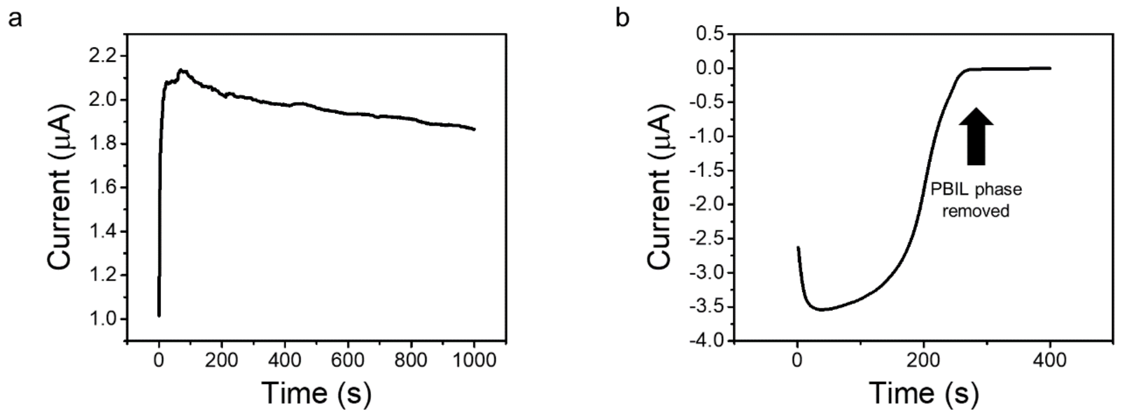

Figure S8. Current response of a 10 μm Pt UME immersed in 0.25 M MEPBr + 0.5 M H_2SO_4 during the PBIL phase formation at 1.1 V (a) and dissolution at 0.9 V (b), corresponding to the Nyquist plots.

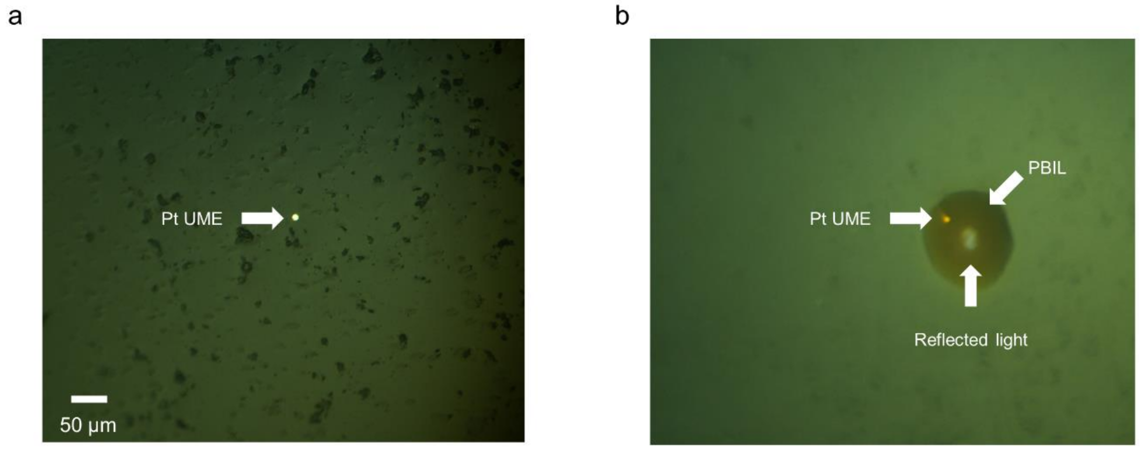

Figure S9. Optical microscope images of a 10 µm Pt UME before (a) and after PBIL formation (b) under the same conditions as in Figure S8a.

Note S3. Electrochemical Raman Spectroscopy for Verification of Presence of Polybromides

The Raman spectroscopic setup is illustrated in **Figure S10a**. A 1 μL droplet of PBIL—prepared in large scale as noted in the Experimental Section (**Figure S2**)—was placed on a Pt wafer, and the wafer was immersed in 0.5 M H_2SO_4 to monitor changes in the Raman spectra under applied potential. Prior to biasing, the Pt electrode potential was pinned at the equilibrium potential of PBIL (approximately 1.0 V vs Ag/AgBr). **Figure S10b** shows the current transient recorded during the application of 0.91 V for 200 s, with the full spectral evolution provided in **Figures S11a** and **S11b**.

The spectra reveal an overall decrease in intensity, attributable to the reduction of Br_{2n+1}^{-} to Br^{-}, which causes shrinkage of the PBIL phase (also evident in **Figure S8b**). In addition, two peaks at 164.5 and 215.7 cm^{-1}, initially not visible in the spectrum, emerge over time. Normalized spectra further show a red shift in the main peak, from 268.1 cm^{-1} to 257.8 cm^{-1} (**Figure S11c**). The initial and final Raman spectra obtained during electrochemical reduction were fitted to the characteristic modes of Br_{2n+1}^{-}s (**Figure S11d**, **e**, **Table S1**).[60–62] The two new peaks at 164.5 and 215.7 cm^{-1} were assigned to the symmetric stretch of Br_3^{-} and asymmetric stretch of Br_5^{-}, respectively. When the normalized intensities of the Gaussian-fitted peaks were plotted against the normal modes of Br_{2n+1}^{-}s (**Figure S12**), the results indicated a population shift toward lower-order Br_{2n+1}^{-}s upon reduction. Overall, the Raman spectroscopic analysis confirms that the oily orange phase contains polybromide ions, and its composition varies dynamically with potential. Specifically, reduction (corresponding to cell discharging) drives the population toward lower-order Br_{2n+1}^{-} species, whereas oxidation (charging) would shift the population toward higher-order species.

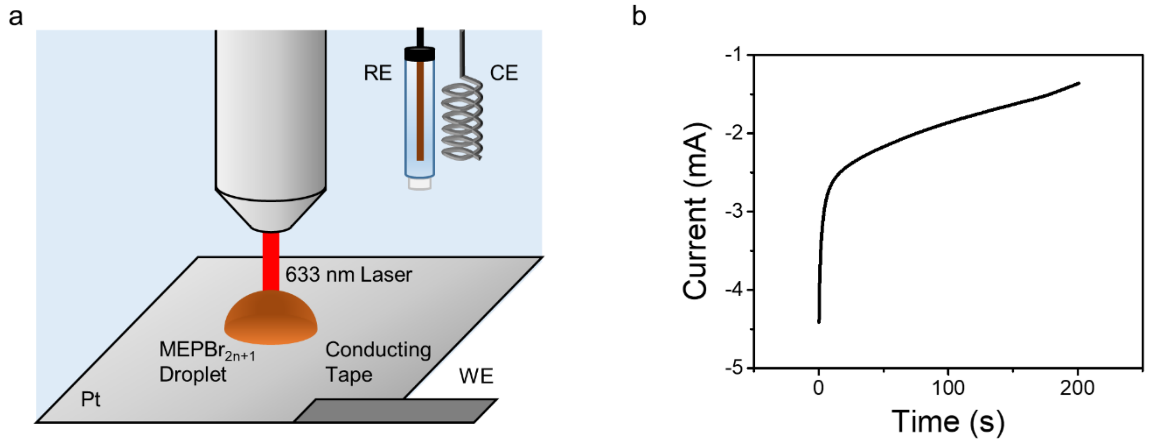

Figure S10. (a)Schematic visualization of the electrochemical Raman spectroscopy experimental setup. (b) Current profile during the application of 0.91 V to a PBIL droplet immersed in 0.5 M H_2SO_4 for 200 s.

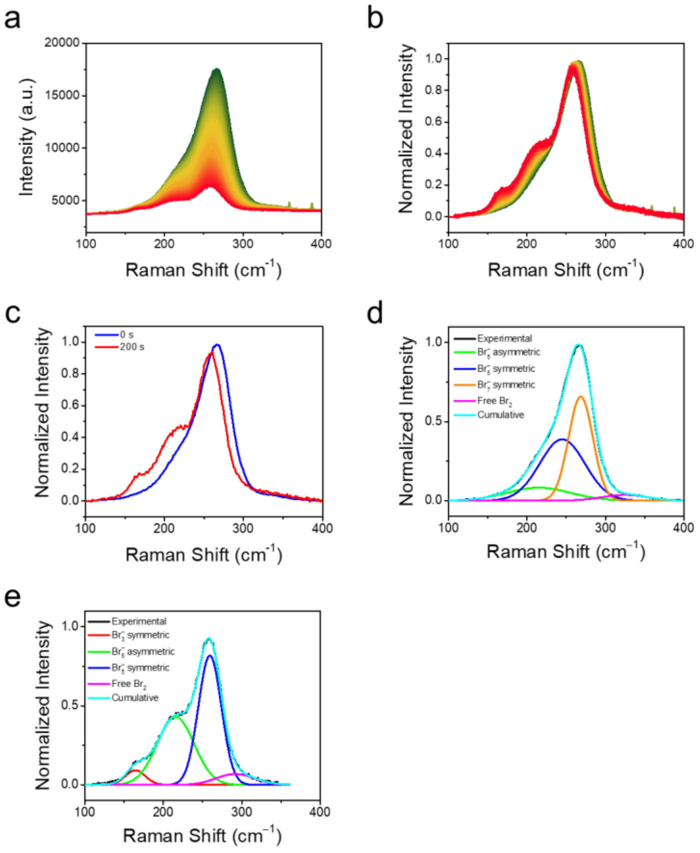

Figure S11. Full Raman spectral transition during 200 s of electrochemical reduction under the conditions described in Figure S10, shown in terms of (a) intensity and (b) normalized intensity. (c) Normalized Raman spectra of PBIL immersed in 0.5 M H_2SO_4, and PBIL after 200 s of reduction. Gaussian peak fitting of Raman spectra presented in (c), recorded (d) before and (e) after electrochemical reduction.

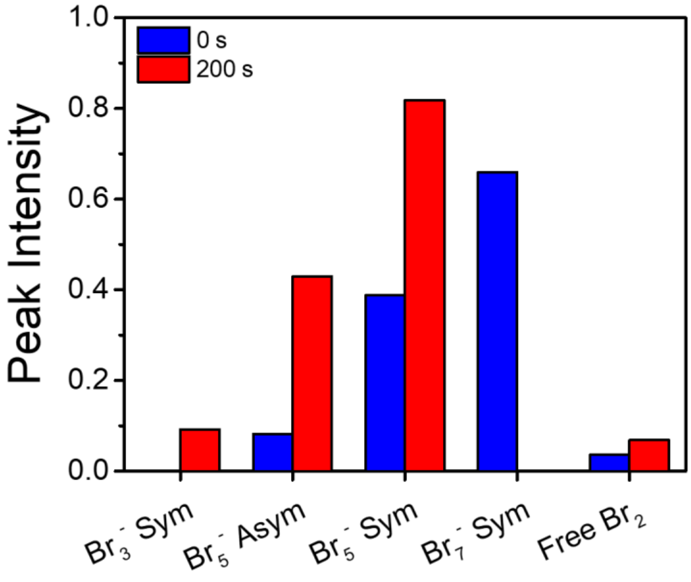

Figure S12. Intensity of Raman peaks fitted from the normalized Raman spectra for PBIL immersed in 0.5 M H_2SO_4, and PBIL after 200 s of reduction.

Table S1. List of Raman peak positions observed in this study compared to those reported in the literature.

	Expected peak position (cm^{-1})					
	0 s	200s	Ref 1 Exp.[60]	Ref 1 Calc.[60]	Ref 2 Exp.[61]	Ref 3 Exp.[62]
Br_3^- Symmetric		164.5	163	171		170
Br_5^- Asymmetric	215.0	215.7	210	217	214	220
Br_5^- Symmetric	244.9	259.4	253	252	249	258
Br_7^- Symmetric	268.5		270	282	269	269
Free Br_2	324.5	291.6			296-302	

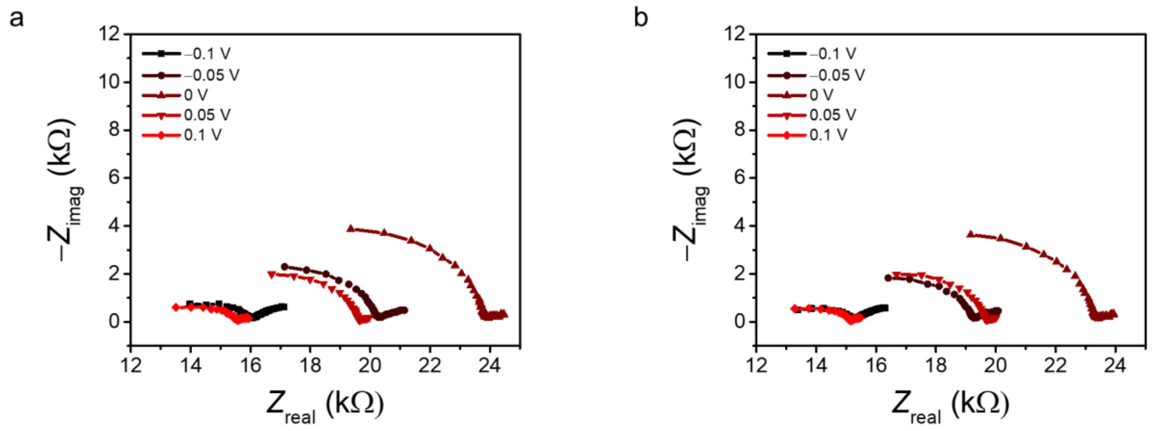

Figure S13. Nyquist plots at selected overpotentials for (a) 0.5 M H_2SO_4 and (b) 0.5 M H_2SO_4 + 0.1 M MEPBr, recorded during the cyclic voltammogram shown in Figure 3b.

Note S4. Derivation of Spherical Warburg Impedance Expression Versus Overpotential

The rigorous way of fitting the impedance data is to use the impedance expression when molecules are diffusing to a disk. However, as mentioned in the reference,[44] the expression is extremely complicated as it is a form of integration of a first order, first kind Bessel function. For the sake of simplicity and improved parameter fitting, we established our logical framework based on a spherical diffusion Warburg, which served as the foundation for the subsequent analysis and fitting process.

When a potential (E) is applied to an electrode immersed in a solution containing a redox pair, O and R, we can consider the following reaction,

$$\mathrm{O} + n\mathrm{e}^- \rightleftarrows \mathrm{R} \tag{S1}$$

The potential dependent spherical diffusion Warburg element expression when the electrode geometry is hemispherical is derived on our previous work:[44]

$$Z_{\mathrm{W,sph,O}} = \frac{RT}{n^2F^2A\sqrt{D_\mathrm{O}}\left(\sqrt{j\omega}+\frac{\sqrt{D_\mathrm{O}}}{r_0}\right)} \cdot \frac{1}{\left(\alpha C_\mathrm{O}(r_0,t)+(1-\alpha)e^{nf(E-E^{0\prime})}C_\mathrm{R}(r_0,t)\right)} \tag{S2}$$

$$Z_{\mathrm{W,sph,R}} = \frac{RT}{n^2F^2A\sqrt{D_\mathrm{R}}\left(\sqrt{j\omega}+\frac{\sqrt{D_\mathrm{R}}}{r_0}\right)} \cdot \frac{e^{nf(E-E^{0\prime})}}{\left(\alpha C_\mathrm{O}(r_0,t)+(1-\alpha)e^{nf(E-E^{0\prime})}C_\mathrm{R}(r_0,t)\right)} \tag{S3}$$

$$Z_{\mathrm{W,sph}} = Z_{\mathrm{W,sph,O}} + Z_{\mathrm{W,sph,R}} \tag{S4}$$

when $E^{0\prime}$ is the standard potential, k^0 is the standard rate constant, $f = F/RT$, α is the transfer coefficient, A is the electrode area, ω is the angular frequency, D is the diffusion coefficient, C is the concentration, and r_0 is the radius of the electrode. Since we are employing a hemispherical diffusion model, $C(r_0, t)$ represents the surface concentration at time t. For simplicity, we will denote it as $C(0)$ moving forward, where z = 0, while also omitting the explicit dependence on time t. O and R represent the oxidized and reduced species, respectively. When plugging in $E_\mathrm{eq} = E^{0\prime} + \frac{RT}{nF}\ln\frac{C_\mathrm{O}^*}{C_\mathrm{R}^*}$, we obtain:

$$Z_{\mathrm{W,sph,O}} = \frac{RT}{n^2F^2A\sqrt{D_\mathrm{O}}\left(\sqrt{j\omega}+\frac{\sqrt{D_\mathrm{O}}}{r_0}\right)} \cdot \frac{C_\mathrm{R}^*}{\left(\alpha C_\mathrm{O}(0)C_\mathrm{R}^*+(1-\alpha)e^{nf\eta}C_\mathrm{R}(0)C_\mathrm{O}^*\right)} \tag{S5}$$

$$Z_{\mathrm{W,sph,R}} = \frac{RT}{n^2F^2A\sqrt{D_\mathrm{R}}\left(\sqrt{j\omega}+\frac{\sqrt{D_\mathrm{R}}}{r_0}\right)} \cdot \frac{e^{nf\eta}C_\mathrm{O}^*}{\left(\alpha C_\mathrm{O}(0)C_\mathrm{R}^*+(1-\alpha)e^{nf\eta}C_\mathrm{R}(0)C_\mathrm{O}^*\right)} \quad \text{(S6)}$$

when $\eta=E-E_{\mathrm{eq}}$ and C^* is the bulk concentration.

For η << 0 and $C_\mathrm{R}^* \sim C_\mathrm{O}^*$, the condition $C_\mathrm{R}^* \gg e^{nf\eta}C_\mathrm{O}^*$ holds true. Additionally, as long as $\sqrt{D_\mathrm{O}} \sim \sqrt{D_\mathrm{R}}$, we can assume that $Z_{\mathrm{W,sph,O}}$ >> $Z_{\mathrm{W,sph,R}}$, leading to $Z_{\mathrm{W,sph}} \approx Z_{\mathrm{W,sph,O}}$. Then we can write:

$$Z_{\mathrm{W,sph}} \approx \frac{RT}{n^2F^2A\sqrt{D_\mathrm{O}}\left(\sqrt{j\omega}+\frac{\sqrt{D_\mathrm{O}}}{r_0}\right)} \cdot \frac{C_\mathrm{R}^*}{\left(\alpha C_\mathrm{O}(0)C_\mathrm{R}^*+(1-\alpha)e^{nf\eta}C_\mathrm{R}(0)C_\mathrm{O}^*\right)} = \frac{\sigma_{\mathrm{Red}}}{\sqrt{j\omega}+\frac{\sqrt{D_\mathrm{O}}}{r_0}} \quad \text{(S7)}$$

when

$$\sigma_{\mathrm{Red}} = \frac{RTC_\mathrm{R}^*}{n^2F^2A\sqrt{D_\mathrm{O}}\left(\alpha C_\mathrm{O}(0)C_\mathrm{R}^*+(1-\alpha)e^{nf\eta}C_\mathrm{R}(0)C_\mathrm{O}^*\right)} \quad \text{(S8)}$$

denotes the Warburg coefficient σ for reducing potential.

For η >> 0, $Z_{\mathrm{W,sph}} \approx Z_{\mathrm{W,sph,R}}$ with similar assumption:

$$Z_{\mathrm{W,sph}} \approx \frac{RT}{n^2F^2A\sqrt{D_\mathrm{R}}\left(\sqrt{j\omega}+\frac{\sqrt{D_\mathrm{R}}}{r_0}\right)} \cdot \frac{e^{nf\eta}C_\mathrm{O}^*}{\left(\alpha C_\mathrm{O}(0)C_\mathrm{R}^*+(1-\alpha)e^{nf\eta}C_\mathrm{R}(0)C_\mathrm{O}^*\right)} = \frac{\sigma_{\mathrm{Ox}}}{\sqrt{j\omega}+\frac{\sqrt{D_\mathrm{R}}}{r_0}} \quad \text{(S9)}$$

when

$$\sigma_{\mathrm{Ox}} = \frac{RTe^{nf\eta}C_\mathrm{O}^*}{n^2F^2A\sqrt{D_\mathrm{R}}\left(\alpha C_\mathrm{O}(0)C_\mathrm{R}^*+(1-\alpha)e^{nf\eta}C_\mathrm{R}(0)C_\mathrm{O}^*\right)} \quad \text{(S10)}$$

denotes the Warburg coefficient σ for oxidizing potential.

When the system is in a charge transfer-limited regime, the surface concentration can be assumed to not differ significantly from the bulk concentration, allowing the Warburg coefficient to be simplified by assuming $C(0) \sim C^*$. This assumption leads to the condition $C_\mathrm{O}(0)C_\mathrm{R}^* \sim C_\mathrm{O}^*C_\mathrm{R}(0)$, with further simplifications possible under the condition $|\eta|$ >> 0:

$$\sigma_{\mathrm{Red}} = \frac{RT}{n^2F^2A\sqrt{D_\mathrm{O}}\left(\alpha C_\mathrm{O}(0)\right)} \quad \text{(S11)}$$

$$\sigma_{\mathrm{Ox}} = \frac{RT}{n^2F^2A\sqrt{D_\mathrm{R}}\left((1-\alpha)C_\mathrm{R}(0)\right)} \quad \text{(S12)}$$

Note S5. Quantification of Polybromide (or Bromine) and Bromide confined in PBIL phase

A 2 μL sample of PBIL was collected and placed on a Pt wafer, which was immersed in 1 mL of 0.5 M H_2SO_4 (**Figure S14a**). A potential of 0.7 V vs. 3 M Ag/AgBr was applied to electrochemically reduce the droplet until the current approached zero (**Figure S14b**), assuming that the majority of the bromine (polybromide) remained confined within the PBIL phase before electrochemical reduction due to their low solubility in water. From the total charge passed, the amount of Br_2 confined in the PBIL phase was calculated, considering Br_{2n+1}^- as an adduct of n Br_2 molecules with a single Br^-. Since 2 moles of electrons are required to reduce 1 mole of Br_2, the concentration can be calculated as:

$$C_{\mathrm{Br_2}} = \frac{n\ \mathrm{mol\ Br_2}}{2\ \mu\mathrm{L}} = \frac{\text{(Passed Charge)}}{2F \times 2 \cdot 10^{-6}\ \mathrm{L}}$$

After the PBIL was fully reduced, a silver wire deposited with AgBr was immersed, and the open circuit potential (V_{OCP}) was measured against the 3 M Ag/AgBr reference electrode (**Figure S14c**). From V_{OCP}, the concentration of Br^- in the 1 mL H_2SO_4 solution was calculated. Using this, the total concentration of bromine atoms originally present in the PBIL droplet (C'_{Br}) was determined using the Nernst Equation:

$$V_{\mathrm{OCP}} = \frac{RT}{2F} \ln \frac{1}{C'_{\mathrm{Br}} \times 2\ \mu\mathrm{L}/1\ \mathrm{mL}} - \frac{RT}{2F} \ln \frac{1}{3}$$

Since C'_{Br} accounts for the total bromine atoms in Br_2 and those existing as Br^-, the concentration of Br^- originally present in the PBIL droplet can be calculated by subtracting $2C_{\mathrm{Br_2}}$ from C'_{Br}:

$$C_{\mathrm{Br^-}} = C'_{\mathrm{Br}} - 2C_{\mathrm{Br_2}}$$

Table S2 summarizes the calculated concentration values. As stated in the main text, the "concentration of Br_{2n+1}^{-}" is defined in **Equation 8** as the concentration-weighted sum of Br_2 units contained in all polybromide anions:

$$C'_{\mathrm{Br}_{2n+1}^{-}} = \sum_{n \geq 1} n C_{\mathrm{Br}_{2n+1}^{-}}.$$

By construction, this quantity is numerically identical to the effective Br_2 concentration, C_{Br_2}. Accordingly, the value of C_{Br_2} determined above can be used directly as the equivalent polybromide concentration, $C'_{\mathrm{Br}_{2n+1}^{-}}$.

The concentrations obtained from these electroanalytical methods carry relatively large errors, as accurately dispensing PBIL liquid is challenging due to its strong hydrophobicity. A micropipette with a plastic tip was used to handle the PBIL, but the liquid did not easily detach from the tip. In addition, the total bromine content was measured by potentiometry, where even a small potential drift leads to a large deviation in the calculated concentration because of its logarithmic dependence. This error from potentiometry is propagated into the estimated polybromide concentration, resulting in substantial uncertainty. As a disclaimer, the reported concentration values were used to verify the assumption of comparable concentration magnitudes, and not for precise quantitative calculations.

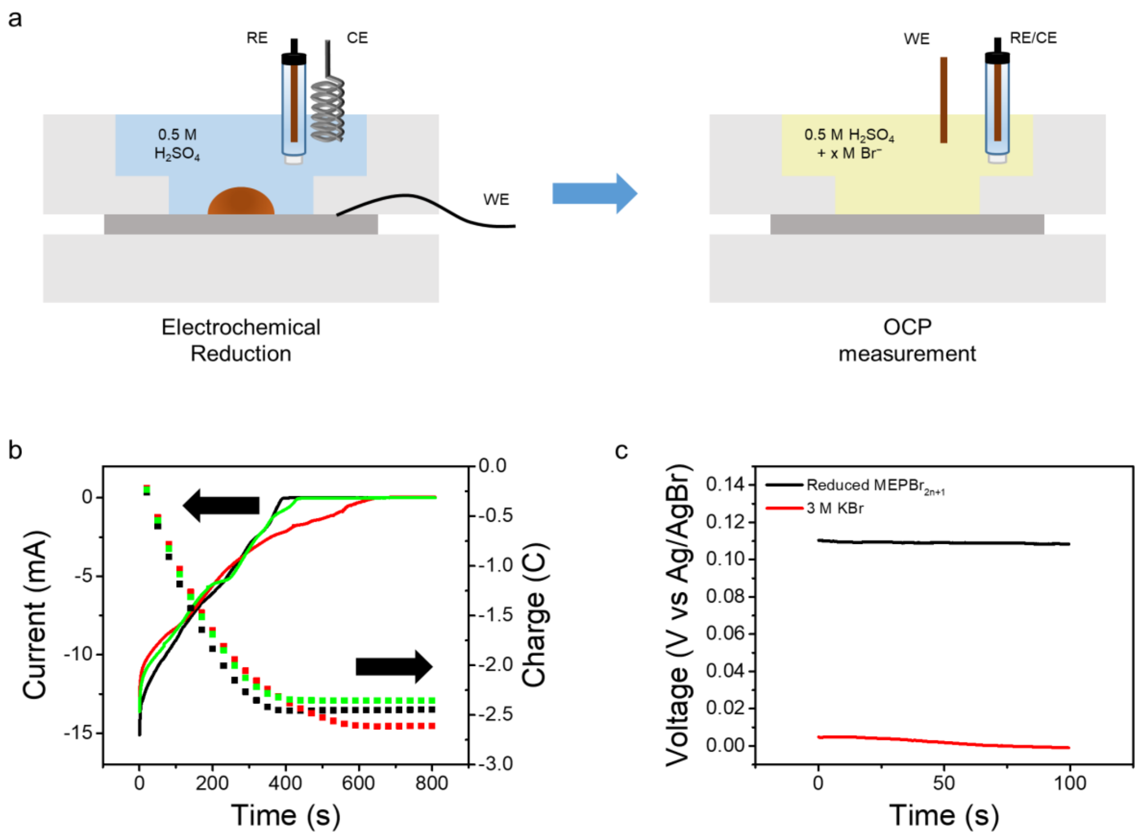

Figure S14. (a) Schematic illustration of the quantification process for bromine species in the synthesized PBIL. (b) Current response (solid line) and passed charge (dotted line) during the full reduction of 2 μL PBIL placed on a Pt wafer by applying 0.7 V vs. 3 M Ag/AgBr over three trials. (c) Chronopotentiometry profile of an Ag/AgBr wire immersed in 1 mL of 0.5 M H_2SO_4 with fully reduced PBIL (black) and in 3 M KBr solution (red)

Table S2. Calculated concentrations of Br_2 and Br^- initially present in 2 μL of PBIL.

Bromine Species	Concentration (M)
Br_2 originally present in PBIL	6.4±0.3
Total bromine atom originally present in PBIL	20±3
Br^- originally present in PBIL	7±3

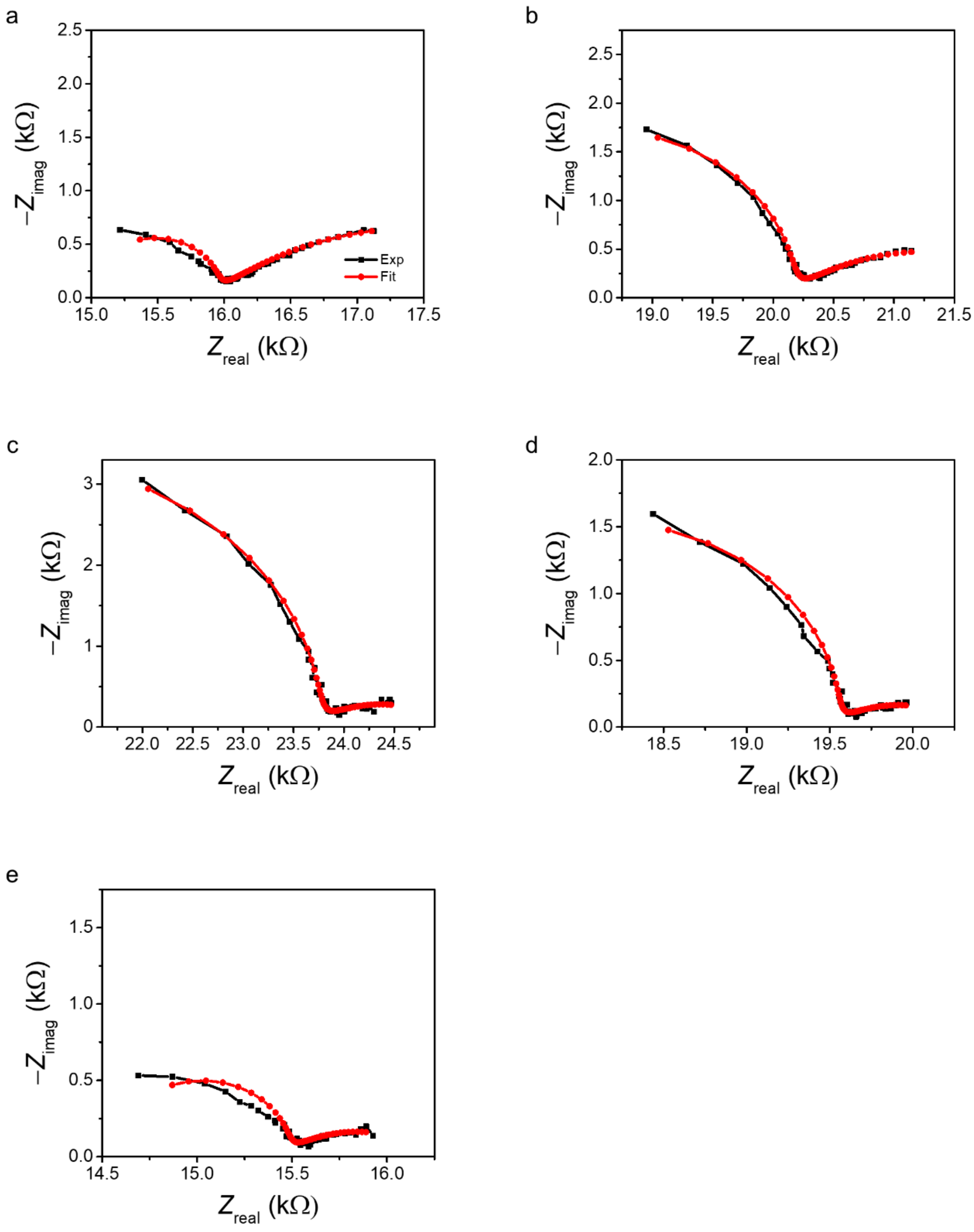

Figure S15. Equivalent circuit fit results of the Nyquist plots shown in Figure S13a, corresponding to overpotentials of (a) −0.1 V, (b) −0.05 V, (c) 0 V, (d) 0.05 V, and (e) 0.1 V.

Note S6. Justification of Selecting the Potential Range for Equivalent Circuit Model Fitting

Unlike the cyclic voltammogram shown in **Figure 3b**, when the overpotential was swept to more positive values (0.18 V), a current hysteresis was observed. This indicates significant changes in the concentration of bromine species near the electrode due to the oxidation reaction, which could be attributed to the accumulation of polybromide or the depletion of bromide (**Figure S16a**). The corresponding Nyquist plots also exhibited anomalous behavior, deviating from a Randles-like Nyquist plot to a two-step diffusion pattern at 0.18 V, accompanied by an increase in overall impedance when reverted back to 0.1 V, further displaying a hysteresis effect (**Figure S16b**).

This behavior was not observed when the potential was cycled up to 0.1 V, either in the Nyquist plots or the cyclic voltammograms (**Figure S16c**). Therefore, we selected 0.1 V as the upper bound for further study. However, to apply the fitting model using the two parameters σ and D_{app} (as described in **Equations S9** and **7**), it is necessary to justify that 0.1 V represents a sufficiently large overpotential to neglect the contribution of $Z_{\mathrm{w,sph,O}}$.

The condition for neglecting $Z_{\mathrm{W,sph,O}}$ is $C_{\mathrm{R}}^{*} \ll e^{nf\eta}C_{\mathrm{O}}^{*}$. For $e^{nf\eta} = 10^{3}$, this corresponds to η=0.088 V. Assuming that $C_{\mathrm{R}}^{*} \sim C_{\mathrm{O}}^{*}$, respectively, we can reasonably assume that the condition $C_{\mathrm{R}}^{*} \ll e^{nf\eta}C_{\mathrm{O}}^{*}$ holds for η=0.1 V. Considering the results from compositional analysis that $C_{\mathrm{R}}^{*} < C_{\mathrm{O}}^{*}$ when PBIL is immersed in aqueous solution, $C_{\mathrm{R}}^{*} \ll e^{nf\eta}C_{\mathrm{O}}^{*}$ also holds for η=0.1 V. A similar condition holds for the reduction process: when η=–0.088 V, $e^{nf\eta} = 10^{-3}$, which leads to $C_{\mathrm{R}}^{*} \gg e^{nf\eta}C_{\mathrm{O}}^{*}$, indicating that the overpotential is sufficiently negative to justify neglecting $Z_{\mathrm{W,sph,R}}$.

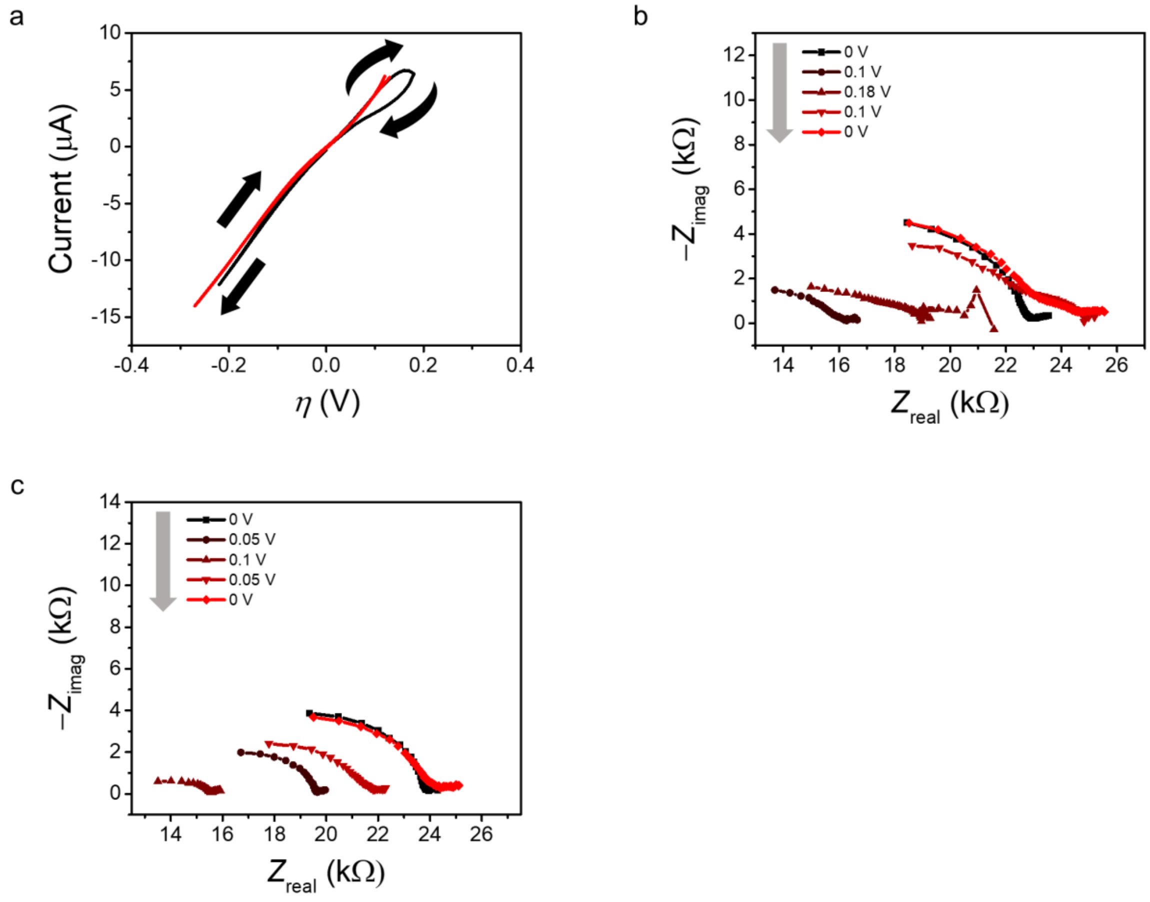

Figure S16. (a) Cyclic voltammogram of 5 µL PBIL placed on a 10 µm Pt UME, cycled up to approximately 0.1 V overpotential (red) and 0.18 V overpotential (black). The black arrows indicate the scan direction during the potential cycling. (b) Nyquist plots at selected overpotentials during the cycling of the black trace in (a). (c) Nyquist plots at selected overpotentials during the cycling of the red trace in (a). Gray arrows next to the legends in (b) and (c) indicate the order of potential cycling.

Note S7. COMSOL Simulation Details and Discussions

COMSOL Multiphysics v5.4, a finite element method simulation software, was used to simulate Nyquist plots for an ultramicroelectrode under the electrodeposition conditions employed in this study. A 2D axisymmetric space dimension was modeled to represent a single ultramicroelectrode with a radius of 10 μm, and the electroanalysis interface was utilized for simulating the Nyquist plots. To construct the model, a 2D axisymmetric space dimension was employed. A quarter-circle geometry was used to simulate a hemispherical space sufficiently larger than the 10 μm radius of the ultramicroelectrode, representing the PBIL phase.

In the simulation, the oxidized species (Ox) was modeled as the molecular bromine, Br_2, whose concentration corresponds to the weighted sum, $C'_{\mathrm{Br}_{2n+1}^-}$, and the reduced species (Red) as the bromide ion, Br^-. The electrochemical reaction was represented as $\mathrm{Ox} + 2\mathrm{e}^- \rightleftharpoons 2\mathrm{Red}$, for representing $\mathrm{Br}_2 + 2\mathrm{e}^- \rightleftharpoons 2\mathrm{Br}^-$, as $C'_{\mathrm{Br}_{2n+1}^-}$ is equivalent to the effective Br_2 concentration. The concentrations of the reduced and oxidized species (C_{Red} and C_{Ox}) were set to 7 M and 6.4 M, respectively, with diffusion coefficients of 1.60×10^{-8} m^2 s^{-1} and 1.27×10^{-9} m^2 s^{-1}, respectively.

The reaction kinetics were modeled using the electroanalytical Butler-Volmer equation. The equilibrium potential was fixed at 0 V, and the electrode potential was scanned over a range from −0.1 V to 0.1 V. The exchange rate constant (i_0) and double-layer capacitance (C_{dl}) were calculated from experimental data as 47,000 A m^{-2} and 1.01 F m^{-2}, respectively. The transfer coefficient (α) was assumed to be 0.55. The simulated Nyquist plots are shown in **Figure S17**.

The Nyquist plots shown in **Figure S17a** exhibit behavior consistent with the experimental results across the overpotential range: the polarization resistance peaks at the equilibrium potential, characteristic of a typical charge transfer-limited system. Furthermore, the mass transport impedance shows pronounced asymmetry, with significantly larger impedance observed at reducing potentials, aligning with the experimental observations.

When the diffusion coefficients of both reduced and oxidized species were fixed at 1.60×10^{-8} m^2 s^{-1} (**Figure S17b**) and 1.27×10^{-9} m^2 s^{-1} (**Figure S17c**), corresponding to the measured values for Br^- and Br_{2n+1}^-, respectively, the polarization resistance still exhibited a maximum near 0 V, consistent with charge transfer-limited behavior. However, the mass-transport asymmetry was either much weaker than in **Figure S17a** or not observed at all. When the bromine species concentrations were reduced by an order of magnitude while maintaining the respective diffusion coefficients of Br^- and Br_{2n+1}^- (**Figure S17d**), diffusion asymmetry was observed, but the system no longer exhibited charge transfer-limited behavior, as the polarization resistance did not display a maximum at η=0 V. These results suggest that the real system may remain charge-transfer-limited across both reductive and oxidative potentials because of the extremely high bromine species concentration, which ensures sufficient supply to the electrode by sheer abundance, despite the relatively low diffusion coefficient of Br_{2n+1}^-.

To ensure the reliability of the simulation, we also simulated the Nyquist plots with ±1 M variations in C_O and C_R (**Figure S18**). The resulting plots showed no significant deviation in trend from those in **Figure S17a**, indicating that uncertainties in concentration have minimal impact on the overall impedance spectra trend.

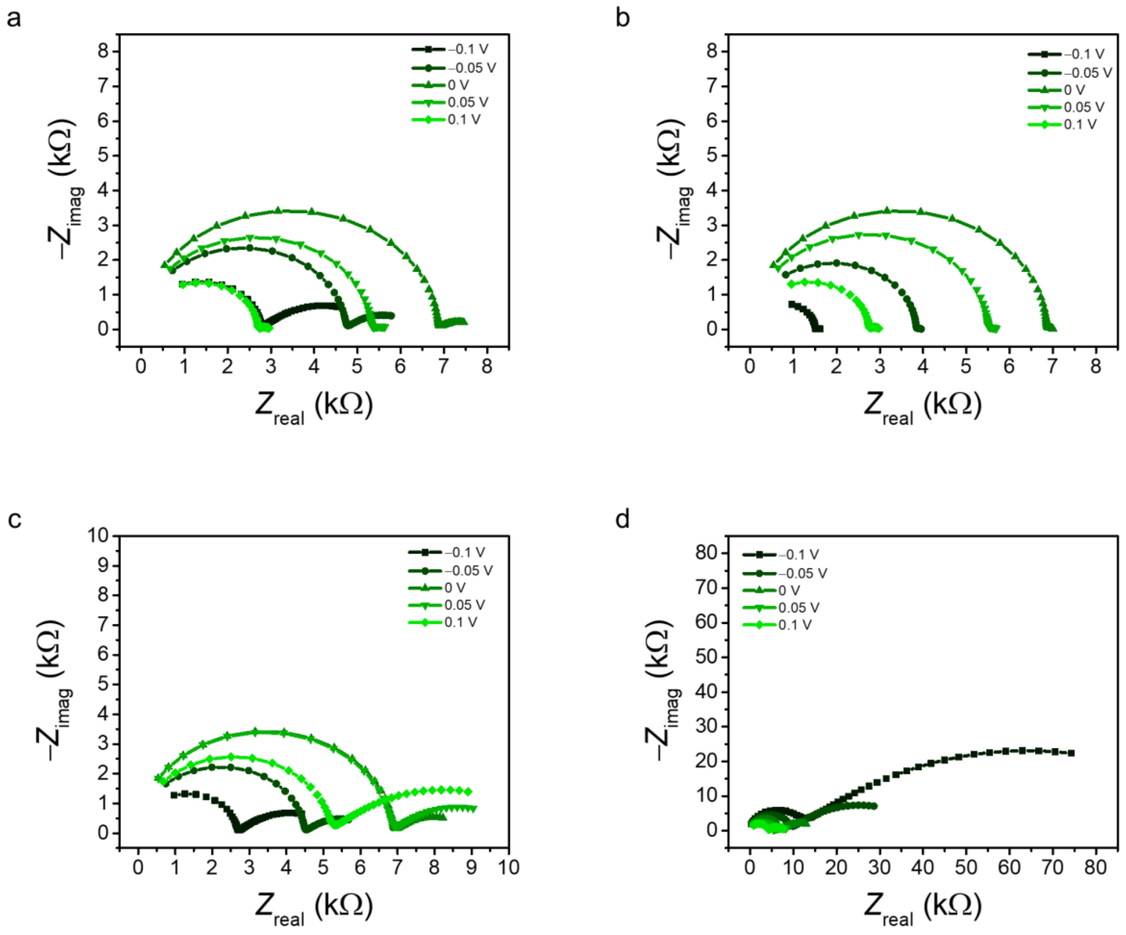

Figure S17. Nyquist plots obtained from COMSOL simulation with the following conditions: (a) D_O=1.27×10^{-9} m^2 s^{-1}, D_R=1.60×10^{-8} m^2 s^{-1}, C_O=6.4 M, C_R=7 M; (b) D_O= D_R=1.60×10^{-8} m^2 s^{-1}, C_O=6.4 M, C_R=7 M; (c) D_O=D_R=1.27×10^{-9} m^2 s^{-1}, C_O=6.4 M, C_R=7 M; (d) D_O=1.27×10^{-9} m^2 s^{-1}, D_R=1.60×10^{-8} m^2 s^{-1}, C_O=0.64 M, C_R=0.7 M.

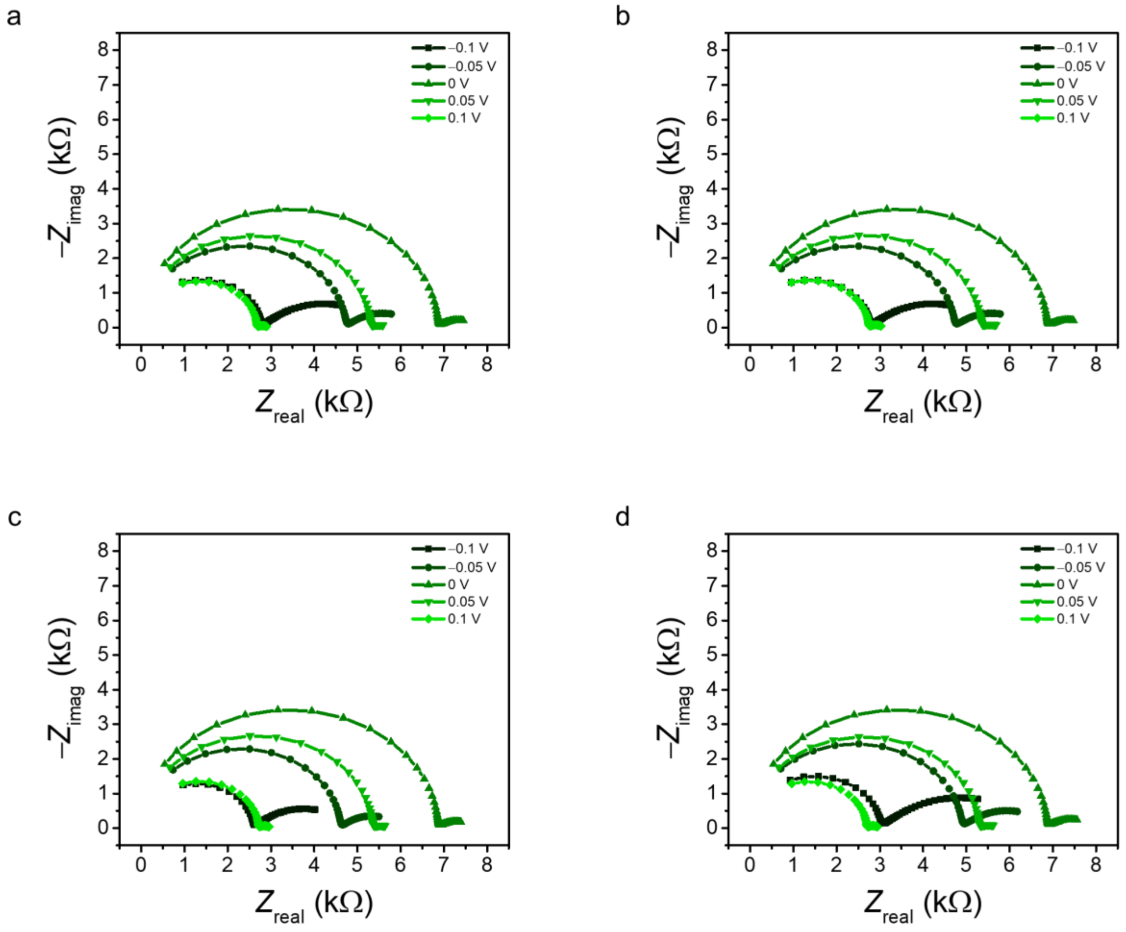

Figure S18. Nyquist plots obtained from COMSOL simulation with the following conditions: (a) C_O=6.4 M, C_R=8 M; (b) C_O=6.4 M, C_R=6 M; (c) C_O=7.4 M, C_R=7 M; (d) C_O=5.4 M, C_R=7 M. The diffusion coefficients were fixed at D_O=1.27×10^{-9} m^2 s^{-1} and D_R=1.60×10^{-8} m^2 s^{-1}.

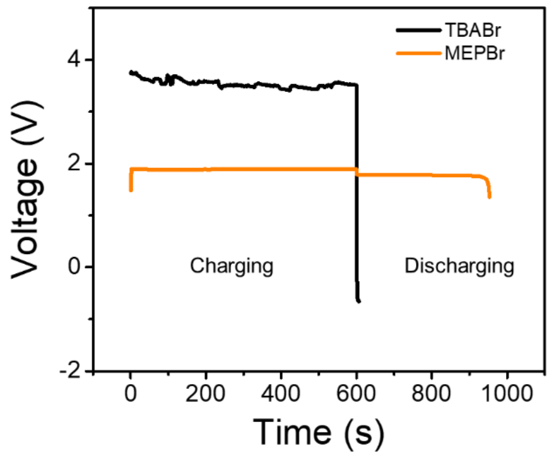

Figure S19. Voltage profiles of dual-plating ZBBs containing 0.5 M $ZnSO_4$ + 0.25 M TBABr (black) and 0.5 M $ZnSO_4$ + 0.25 M MEPBr (orange) during galvanostatic charge/discharge at 102 mA cm^{-2}.

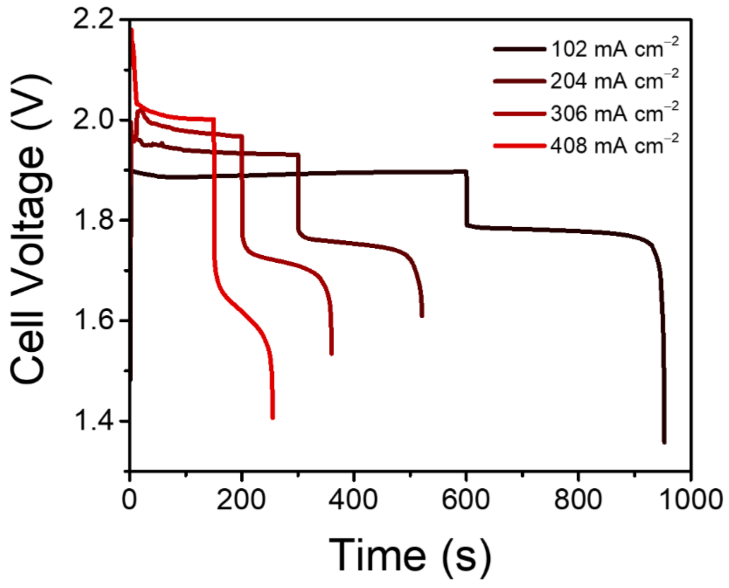

Figure S20. Voltage profile corresponding to the data shown in Figure 4d, plotted against time.

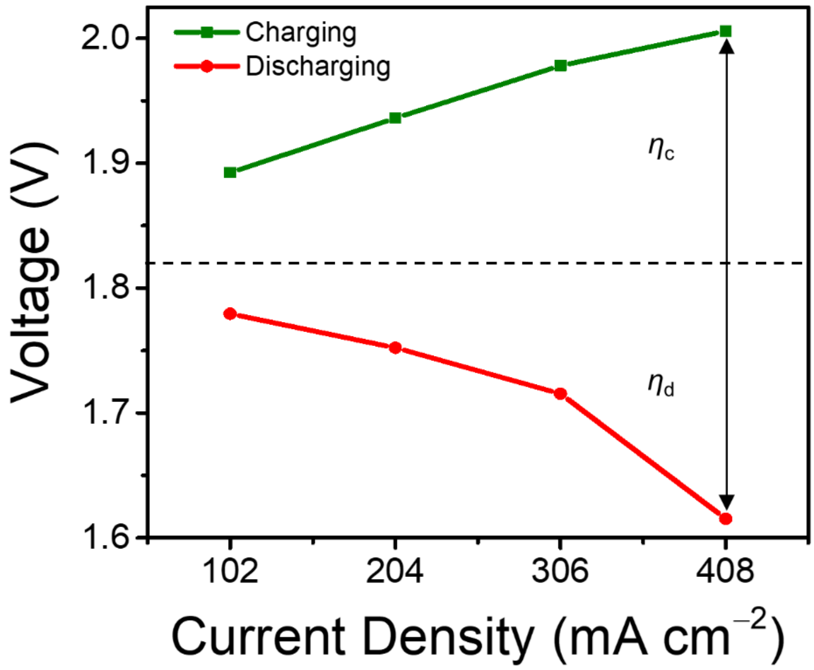

Figure S21. Midpoint potential for each charge/discharge phase in Figure 4d, plotted as a function of current density.

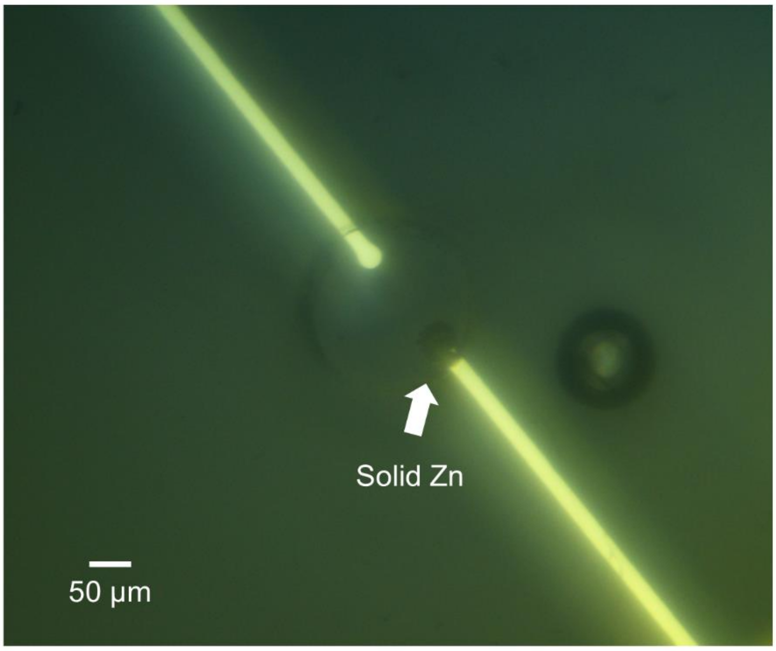

Figure S22. Optical microscope image obtained after four galvanostatic charge–discharge cycles. The positive electrode (top electrode) shows complete removal of PBIL after discharging, whereas the negative electrode (bottom electrode) retains a large amount of deposited zinc.

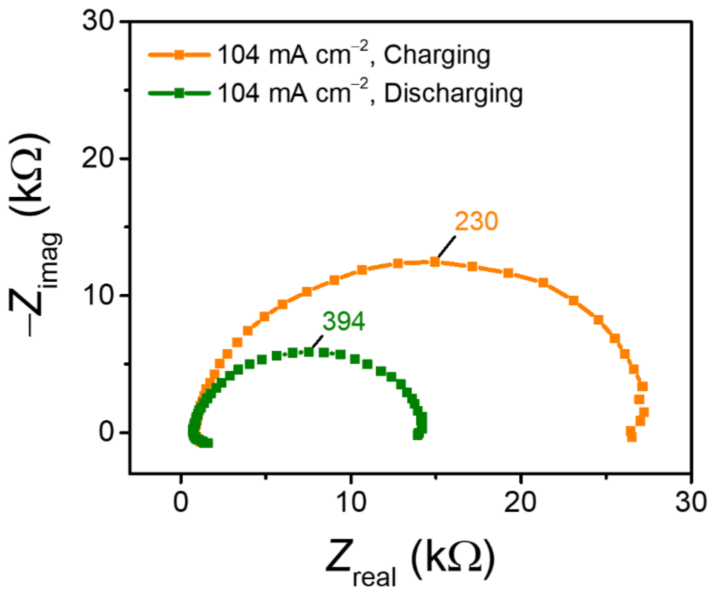

Figure S23. Negative electrode-side Nyquist plots obtained at 102 mA cm^{-2} during charging and discharging (also shown in Figure 4g), with the corresponding characteristic frequencies indicated.

Table S3. Fitted parameters of the Nyquist plots shown in Figure S23 using the equivalent circuit model $R_s(R_pC_{dl})$.

	**102 mA cm^{-2}, Charging**		**102 mA cm^{-2}, Discharging**	
	Fitted Parameters	Relative Standard Error (%)	Fitted Parameters	Relative Standard Error (%)
$\boldsymbol{R_s}$ **(Ω)**	1088	5.4	1057	5.809
$\boldsymbol{R_p}$ **(kΩ)**	25.7	6.867	12.73	7.235
$\boldsymbol{C_{dl}}$ **(nF)**	22.57	6.39	28.23	8.111

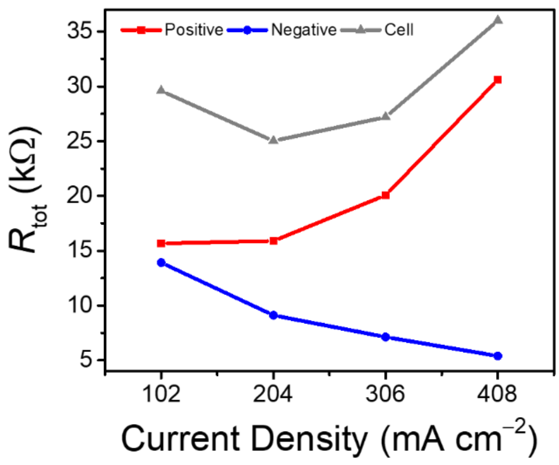

Figure S24. Real part of the impedance at the lowest frequency from the Nyquist plots of the discharge phase in Figure 4g (R_{tot}) for the positive electrode, negative electrode, and full cell, plotted against the corresponding current densities.

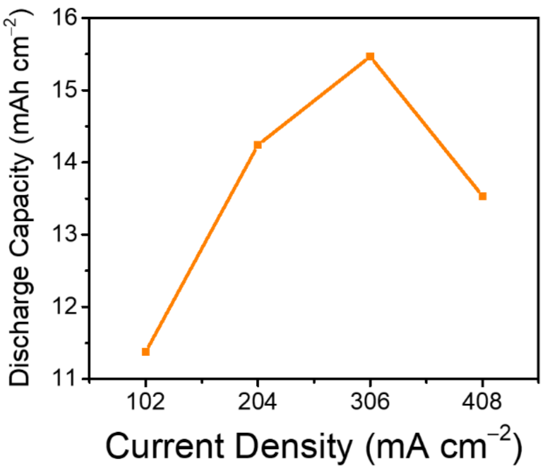

Figure S25. Discharge capacity for each discharge phase in Figure 4d, plotted as a function of current density.

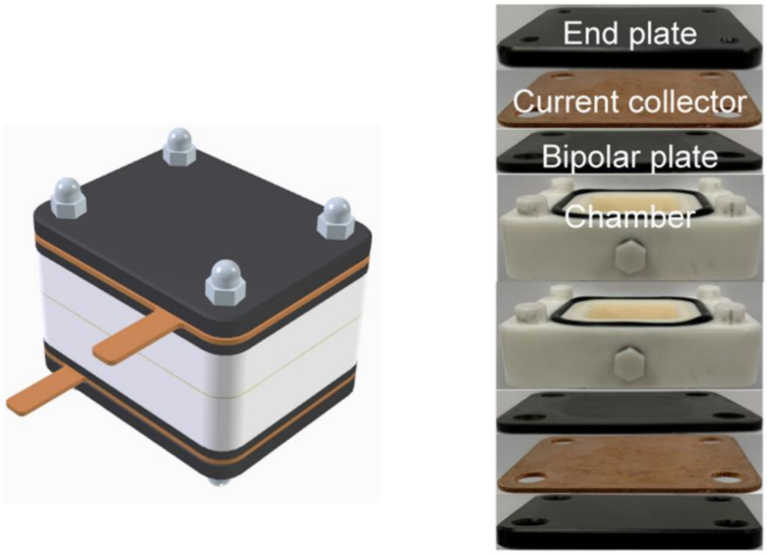

Figure S26. Configuration of the home-made cell used for high areal capacity experiments.

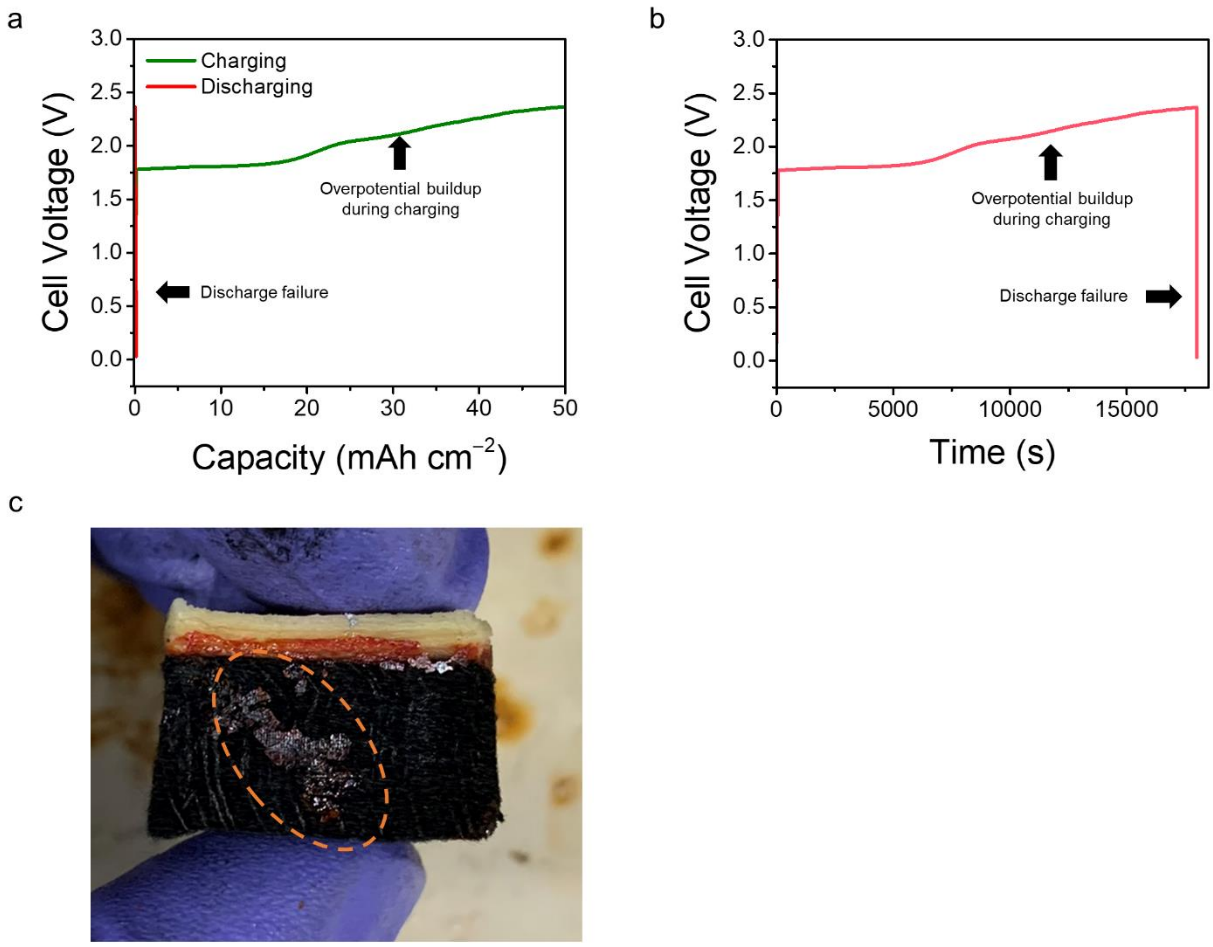

Figure S27. Voltage profile of a dual-plating ZBB during galvanostatic charge/discharge at 10 mA cm^{-2} current density and 50 mAh cm^{-2} charging capacity in an electrolyte containing 2.8 M $ZnBr_2$ and 0.3 M TEABr, plotted against (a) capacity and (b) time. (c) Digital photograph of the $TEABr_{2n+1}$ complex clogging the graphite felt positive electrode, observed during post-mortem analysis.

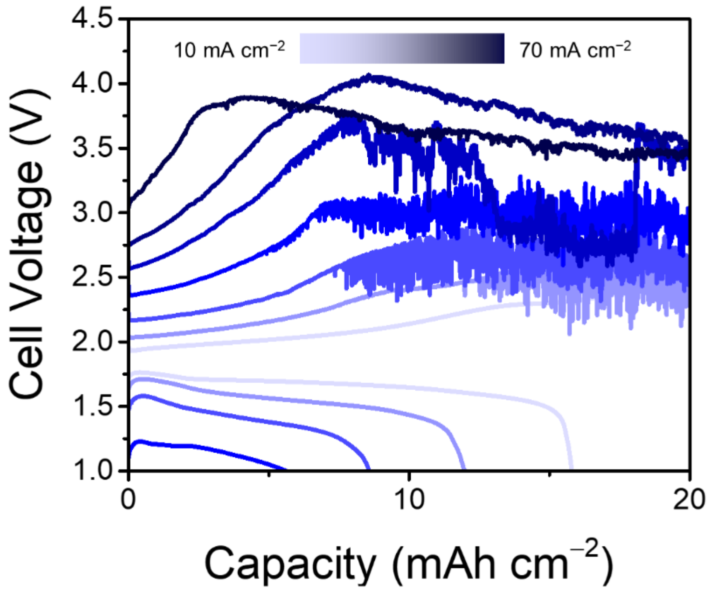

Figure S28. Voltage profile of a dual-plating ZBB during galvanostatic charge/discharge at various current densities and 20 mAh cm^{-2} charging capacity in an electrolyte containing 2.8 M $ZnBr_2$ and 0.1 M TBABr.

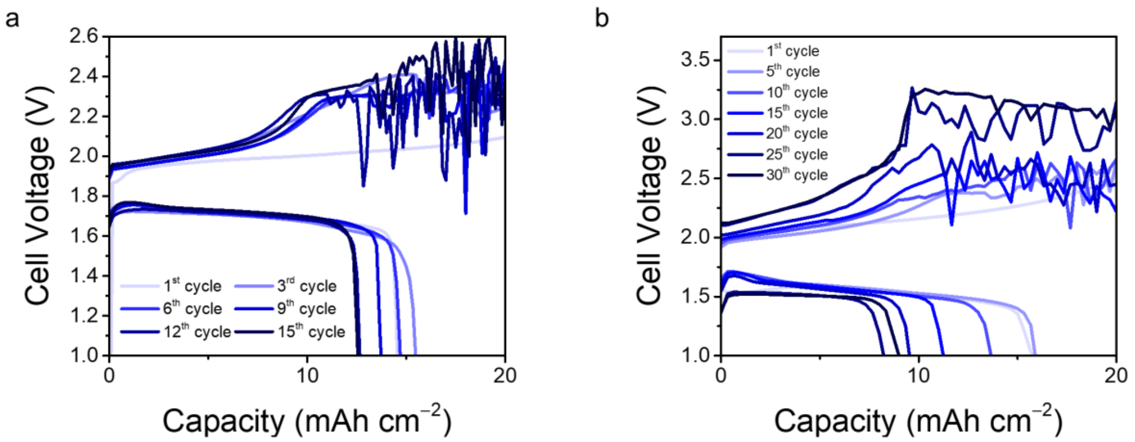

Figure S29. Long-term cycling voltage profile of a dual-plating ZBB during galvanostatic charge/discharge at (a) 10 mA cm^{-2} and (b) 20 mA cm^{-2} with 20 mAh cm^{-2} charging capacity in an electrolyte containing 2.8 M $ZnBr_2$ and 0.1 M TBABr.

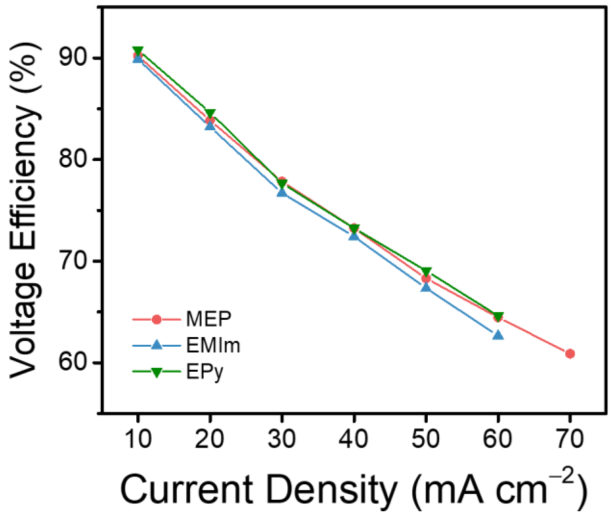

Figure S30. Voltage efficiency (VE) of dual-plating ZBBs utilizing the three IL-forming BCAs—MEPBr, EMImBr, and EPyBr—corresponding to the rate capability tests in Figure 5a.

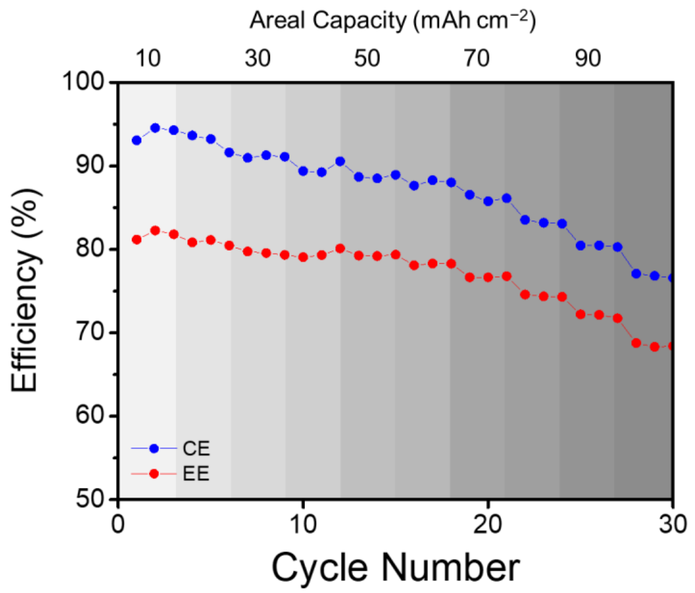

Figure S31. Coulombic efficiency (CE) change with areal capacity variation. The Coulombic efficiency was measured by varying the areal capacity from 10 to 100 mAh cm^{-2} at a fixed current density of 10 mA cm^{-2}. For the experiment, a full cell with 2.8 M $ZnBr_2$, 0.3 M MEPBr was used.
